# Carbon Monoxide Binding to the Iron–Molybdenum Cofactor of Nitrogenase: a Detailed Quantum Mechanics/Molecular Mechanics Investigation

**DOI:** 10.1021/acs.inorgchem.1c02649

**Published:** 2021-11-12

**Authors:** Nico Spiller, Ragnar Bjornsson, Serena DeBeer, Frank Neese

**Affiliations:** † Max-Planck-Institut für Kohlenforschung, Kaiser-Wilhelm-Platz 1, 45470 Mülheim an der Ruhr, Germany; ‡ Max Planck Institute for Chemical Energy Conversion, Stiftstr 34-36, 45470 Mülheim an der Ruhr, Germany

## Abstract

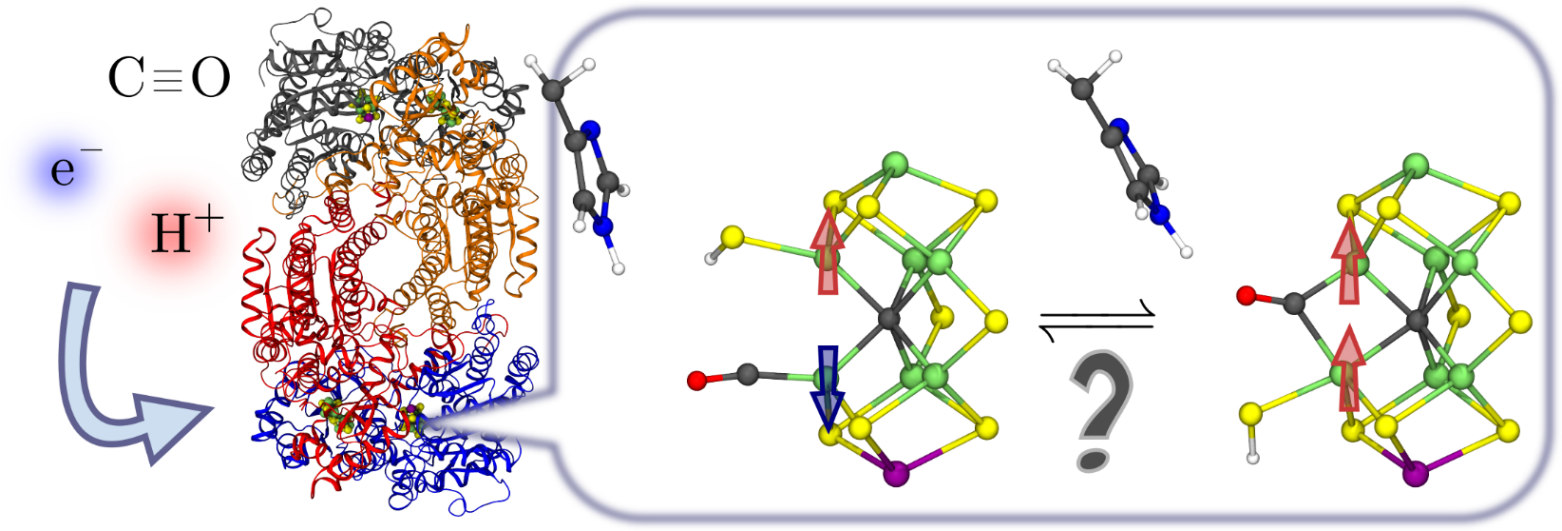

Carbon monoxide (CO) is a well-known inhibitor of nitrogenase activity. Under turnover conditions, CO binds to FeMoco, the active site of Mo nitrogenase. Time-resolved IR measurements suggest an initial terminal CO at 1904 cm^–1^ that converts to a bridging CO at 1715 cm^–1^, and an X-ray structure shows that CO can displace one of the bridging belt sulfides of FeMoco. However, the CO-binding redox state(s) of FeMoco (E_n_) and the role of the protein environment in stabilizing specific CO-bound intermediates remain elusive. In this work, we carry out an in-depth analysis of the CO–FeMoco interaction based on quantum chemical calculations addressing different aspects of the electronic structure. (1) The local electronic structure of the Fe–CO bond is studied through diamagnetically substituted FeMoco. (2) A cluster model of FeMoco within a polarizable continuum illustrates how CO binding may affect the spin-coupling between the metal centers. (3) A QM/MM model incorporates the explicit influence of the amino acid residues surrounding FeMoco in the MoFe protein. The QM/MM model predicts both a terminal and a bridging CO in the E_1_ redox state. The scaled calculated CO frequencies (1922 and 1716 cm^–1^, respectively) are in good agreement with the experimentally observed IR bands supporting CO binding to the E_1_ state. Alternatively, an E_2_ state QM/MM model, which has the same atomic structure as the CO-bound X-ray structure, features a semi-bridging CO with a scaled calculated frequency (1718 cm^–1^) similar to the bridging CO in the E_1_ model.

## Abstract 2

The MoFe protein of wild-type nitrogenase has been shown to bind CO during enzymatic turnover. According to our QM/MM model, the protein environment plays a pivotal role during the binding event. A detailed analysis of the electronic structure reveals that CO modulates the spin-coupling between Fe centers and therefore an atypical set of broken-symmetry determinants has to be considered in the DFT calculations.

## Introduction

Nitrogenases are a group of enzymes that can reduce chemically inert N_2_ to bioavailable ammonia. The best studied class of nitrogenase is Mo nitrogenase.^1^ Here, the proteins required for N_2_ reduction are the Fe protein and the MoFe protein, with the Fe protein functioning as an electron donor to the MoFe protein. Inside the MoFe protein, the electron is transferred *via* the P-cluster, an Fe_8_S_7_ cluster, to the active site, where N_2_ is reduced. The active site is a large iron–sulfur cluster called the iron molybdenum cofactor (FeMoco). It contains seven Fe, a single Mo, and an unusual carbide (C^4–^) as the central atom.^2,3^ The reduction of N_2_ is a complex, little-understood process that requires a total of eight electrons and eight protons being transferred to FeMoco. The individual states in the catalytic cycle are labeled E_n_ according to the Lowe–Thorneley cycle,^4^ where *n* refers to the reduction events relative to the resting state E_0_. The E_4_ state is thought to be the primary redox state that binds N_2_, and the binding event is generally believed to happen as reductive elimination of H_2_ occurs.^5^ The alternative V and Fe nitrogenases contain V or only Fe instead of Mo in their active site,^6^ but all three nitrogenases are believed to follow highly similar N_2_ reduction mechanisms.^7^

**Figure 1:**
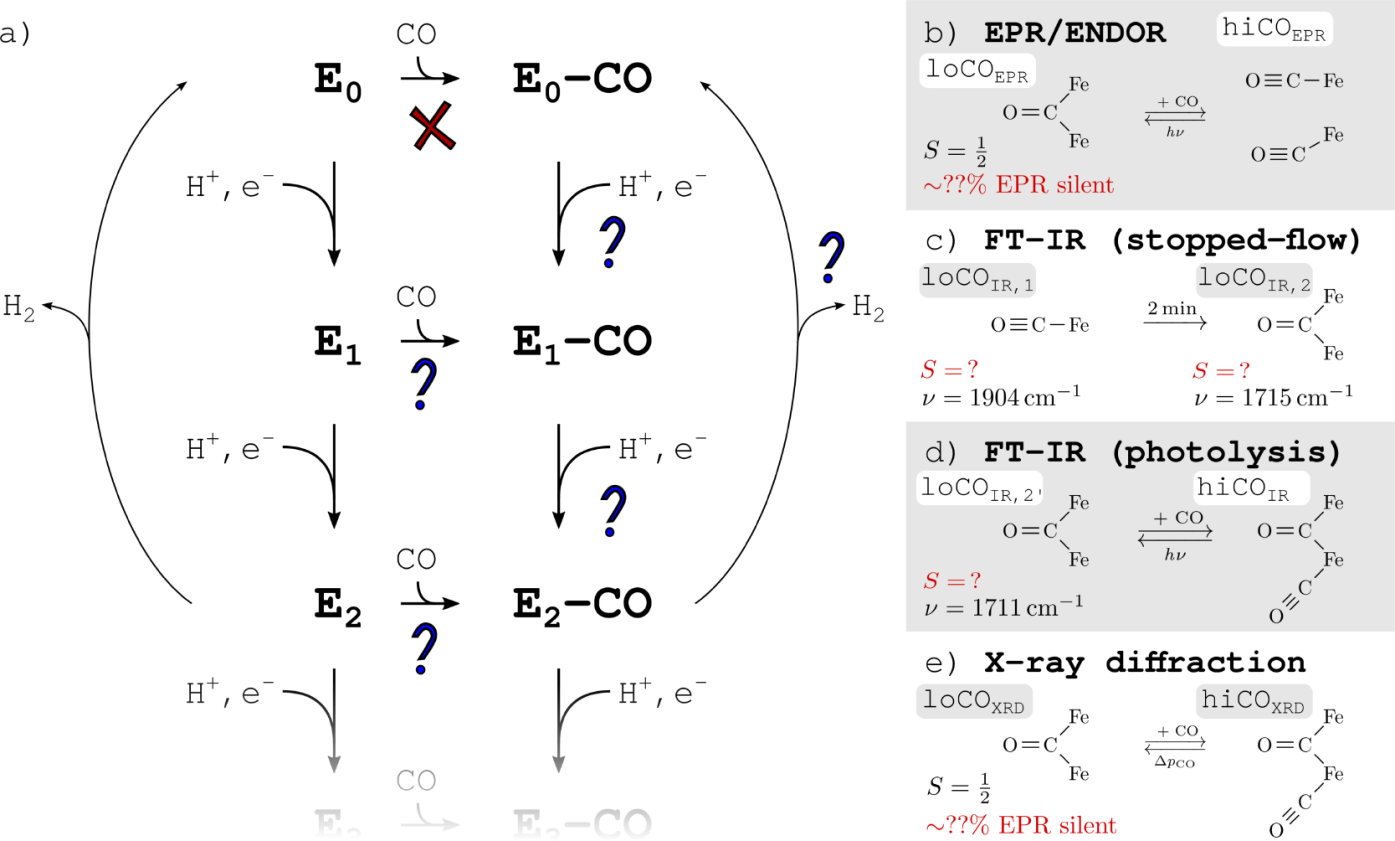
(a) Early E_n_ redox states in the Lowe–Thorneley cycle for nitrogen reduction by Mo nitrogenase and possible CO inhibition. In the resting state of the wild-type MoFe protein (E_0_), the active site FeMoco gives rise to an *S* = 3/2 EPR signal, which is not perturbed in the presence of CO. (b–e) Proposed experimental species with a single CO bound to FeMoco in the MoFe protein: EPR/ENDOR spectroscopy (b), FT-IR spectroscopy (c,d), and X-ray crystallography (e). The single CO species appear under low CO partial pressures and can bind a second CO under high CO partial pressures.

Several other small molecules can act as substrates or inhibitors to nitrogenase such as acetylene, propargyl alcohol, cyanide, or carbon monoxide (CO).^8^ CO is isoelectronic to N_2_ and has been known for several decades to diminish nitrogenase-dependent plant growth.^9^ In the wild-type MoFe protein, CO binds reversibly to the active site and is an inhibitor to N_2_ reduction. Furthermore, it is generally accepted that CO binding in the wild-type MoFe protein requires turnover conditions, that is, the supply of electrons and protons to the active site, and is believed to happen during the early states in the Lowe–Thorneley cycle (E_1_ or E_2_), as illustrated in Figure 1a.^10^ However, V nitrogenase has been reported to bind CO without turnover conditions in the presence of a reductant.^11,12^ Interestingly, V nitrogenase, as well as Fe nitrogenase and Val70 mutants of Mo nitrogenase, have been shown to catalytically reduce CO to hydrocarbons.^13−15^

The experimental techniques that have been employed most often for the study of CO binding to nitrogenase are electron paramagnetic resonance (EPR) and infrared (IR) spectroscopy. With IR spectroscopy, one probes all species irrespective of the E_n_ state. EPR spectroscopy, on the other hand, is typically spin state-selective and routinely probes only even-numbered E_n_ states. While a number of CO-bound IR and EPR species have been reported (*vide infra*), a parallel study of both techniques, which could establish a correspondence between them and further indicate their respective E_n_ state, has not been reported to date. Figure 1b–e gives an overview of the proposed structures based on the experimental observations which are discussed in the following text.

In the resting state E_0_, MoFe-bound FeMoco gives rise to an *S* = 3/2 EPR signal (*g* = [4.33, 3.77, 2.00]).^16^ The charge of FeMoco has been established as [MoFe_7_S_9_C]^1–^,^17−19^ with 41 unpaired electrons being shared among the metal centers (assuming Fe^2+^, Fe^3+^, and Mo^3+^ oxidation states). In the E_1_ state, FeMoco has an even number of electrons, but the spin state—either diamagnetic or integer spin—is currently unknown. It has recently been proposed based on QM/MM calculations that the Fe part of the Mo cubane is reduced in the E_1_ state compared to the E_0_ state.^20,21^ Additionally, multiple computational studies have proposed the belt sulfide S2B or S5A being protonated in the E_1_ state.^20,22−25^ In the E_2_ state, FeMoco exhibits two distinct *S* = 3/2 EPR signals (*g* = [4.21, 3.76, 1.97]/[4.69, 3.20, 2]).^26−29^ Computational models have suggested that hydride formation can occur in this redox state.^22,24,29,30^

The presence of CO does not alter the E_0_ EPR signal of the MoFe protein, which suggests no interaction between CO and FeMoco in the wild-type resting state MoFe protein. However, under turnover conditions, the presence of CO generates two characteristic *S* = 1/2 EPR signals: loCO_EPR_ and hiCO_EPR_, that arise under low and high CO pressures, respectively (*g* = [2.09, 1.97, 1.93]/[2.17, 2.06], labeled lo-CO/hi-CO in the original work).^31^^57^Fe electron nuclear double resonance (ENDOR) measurements on the loCO_EPR_ and hiCO_EPR_ species confirm that CO binds to FeMoco.^32^ The symmetry of the hyperfine coupling tensor extracted from ^13^C-ENDOR spectra leads to the proposition that the loCO_EPR_ species harbors a single bridging μ-CO ligand and hiCO_EPR_ two terminal CO ligands (see Figure 1b).^33^ It is possible to convert the hiCO_EPR_ species to loCO_EPR_ by photolysis, and subsequent annealing restores hiCO_EPR_ with an estimated activation energy of about 1 kcal/mol, implying that loCO_EPR_ and hiCO_EPR_ arise from the same, even-numbered E_n_ state.^34^ Furthermore, the valence assignment based on ^57^Fe ENDOR studies suggest that the loCO_EPR_ and hiCO_EPR_ species, as well as the resting state, have the same formal metal oxidation states.^35^ However, spin quantification for these EPR signals has not been reported for the wild-type MoFe protein but only for the His195→Gln mutant, which gives rise to identical loCO_EPR_ and hiCO_EPR_ signals under comparable conditions. These loCO_EPR_ and hiCO_EPR_ signals have been reported to constitute merely 10 and 26% of the total reaction mixture, respectively, while the resting state *S* = 3/2 signal amounts to 8%.^36^ It has been shown for the His195→Gln mutant that a single CO can be photolysed from a two CO bound species without affecting the EPR spectrum.^37^ Further spin quantification of the EPR spectrum showed a large fraction of FeMoco in an EPR-silent state. These observations suggest that CO binding may also occur at the E_1_ state.

The binding of CO to FeMoco under turnover conditions was followed by time-resolved stopped-flow FTIR (SF-FT-IR) spectroscopy.^38−40^ The electron-flux and CO pressure were comparable to those used in EPR studies (*vide supra*). Under low CO partial pressures, a single transient vibrational band appears at 1904 cm^–1^ (loCO_IR,1_), which is fully converted into a band at 1715 cm^–1^ within 2 min (loCO_IR,2_). The bands were proposed to correspond to a terminal CO that transforms to a bridging μ-CO (see Figure 1c). Under high CO partial pressures, multiple bands were observed between 1700 and 2000 cm^–1^. Two of those bands, at 1906 and 1715 cm^–1^, respectively, closely follow a time course of the 1904 and 1715 cm^–1^ bands at low CO pressures. Therefore, the loCO_IR,1_ and loCO_IR,2_ species observed under low CO pressures are very likely present under high CO pressures as well. The SF-FT-IR experiment with high CO partial pressures was repeated for Val70→Ile and Val70→Gly mutants.^40^ With the more bulky Ile residue, the frequency of the loCO_IR,1_ species shifts from 1906 cm^–1^ (wild-type) to 1895 cm^–1^ and with the spatially less demanding Gly to 1911 cm^–1^. It is of interest to note that CO inhibits the reduction of azide by Mo nitrogenase within less than 400 ms and therefore happens faster than the appearance of the loCO_IR,1_ signal.^41^

The multitude of species present under high CO pressures were characterized further by IR-monitored photolysis.^37,42^ Particularly, the photolysis of hiCO_IR_ to loCO_IR,2′_ (Hi-1 and Lo-1 in the original work) is related to the IR species already discussed (see Figure 1d). hiCO_IR_ exhibits two frequencies that are consistent with a terminal CO and a bridging μ-CO. After photolysis to loCO_IR,2′_, a band at 1711 cm^–1^ indicates a bridging CO, while a second signal corresponds to an unbound CO molecule trapped in some protein pocket. Considering the similar frequencies, the species loCO_IR,2′_ most likely corresponds to the SF-FT-IR species loCO_IR,2_ (1715 cm^–1^). Annealing of loCO_IR,2′_ leads to the recovery of the hiCO_IR_ bands. The estimated activation energy for this conversion of about 1 kcal/mol is in good agreement with the reversible conversion between the EPR species loCO_EPR_ and hiCO_EPR_, suggesting that the detached CO is trapped in a similar protein pocket.

Furthermore, CO binding has also been reported for solution-extracted FeMoco following electrochemical reduction.^43−45^ Here, the authors observed no interaction of CO with solvated FeMoco in an oxidation state showing an *S* = 3/2 EPR signal and therefore most likely corresponding to the E_0_ state of the MoFe protein. However, after one-electron reduction, a single IR band at 1835 cm^–1^ is observed under low CO pressures, which is replaced by a band at 1808 cm^–1^ upon further reduction. Based on the low frequency, the authors interpret both signals as arising from a bridging CO. Also, cyanide has been shown to enable CO binding to the supposedly E_0_-like redox state of solvated FeMoco.^45^

An X-ray diffraction (XRD) structure of the MoFe protein has been solved after putting the system under turnover with low CO partial pressures.^46^ In this 1.5 Å X-ray structure (loCO_XRD_), a μ-CO was found to replace the S2B belt sulfide, which bridges Fe2 and Fe6 in the resting state (see Figure 1d). Such a structure is consistent with a bridging CO that has been proposed for *S* = 1/2 EPR species loCO_EPR_ and the SF-FT-IR species loCO_IR,2_. More recently, a 1.33 Å X-ray structure was obtained by exposing the loCO_XRD_ crystals to high CO pressures.^47^ The resulting X-ray structure (hiCO_XRD_) is actually a superposition of the singly and the doubly CO-bound cofactors. An EPR spectrum shows the presence of loCO_EPR_ for the low-pressure samples and a mixture of loCO_EPR_ and hiCO_EPR_ for the high-pressure samples. Nevertheless, an unambiguous correspondence between the XRD and EPR species cannot be firmly established, as spin quantification of the EPR signals was not reported and hence possible contributions from EPR-silent states are unaccounted for.

Multiple structures of a singly CO-bound FeMoco have been proposed based on density functional theory (DFT) calculations. Rod and Nørskov proposed terminal CO binding to either Fe2, Fe3, or Fe4 based on a FeMoco cluster model and found significantly stronger binding for an E_1_- and an E_2_-type model as compared to E_0_.^48^ Dance suggested the possibility that the vibration of terminal CO is coupled to the Fe–H stretching of a hydride bound to the same Fe center.^49^ The above-mentioned studies, however, assumed either no central atom or a nitride (N^3–^), but not a carbide (C^4–^), as determined later experimentally.^2,3^ Varley and Nørskov proposed a mechanism for CO reduction to methane by an isolated FeMoco model featuring the central carbide.^50^ According to a cluster model by Scott *et al.*, the experimental SF-FT-IR species hiCO_IR_ arises from an E_2_-type cofactor with one terminal CO and one terminal formyl (HCO) species.^51^ With the exception of the QM/MM model used in the quantum refinement of the X-ray structure loCO_XRD_,^52^ none of the computational studies of the CO-FeMoco interaction to date have explicitly included the protein environment. However, the importance of the protein environment has been demonstrated by a number of experimental studies which report distinct CO binding characteristics for multiple MoFe protein mutants.^37,40,42,51,53^

While N_2_ reduction is believed to follow highly similar mechanisms for all three nitrogenases (Mo, V, and Fe),^7^ they exhibit significant differences for CO as a substrate/inhibitor, as explained above.^11−15,54^ In wild-type Mo nitrogenase, CO merely inhibits the catalytic activity, which allows for a cleaner interpretation of the available experimental data. Therefore, in this study, we will focus on the CO-bound intermediates in the wild-type MoFe protein. Within the present study, we would like to address the following questions: (1) What oxidation and protonation states of the cofactor are required for CO binding? (2) What is the initial binding site of CO? (3) How does the protein environment affect the binding of CO? To this end, we examine different E_n_ states, different binding sites (Fe2 and Fe6), and different models for the environment (dielectric continuum *vs* explicit QM/MM). The first part of this study focuses on the local electronic structure of the Fe–CO bond. The second part deals with the complete cofactor in the resting state E_0_. A parallel investigation of a cluster model and a QM/MM model shows the explicit effects of the protein environment on CO binding. The third part focuses on the more reduced E_1_ and E_2_ models. Finally, the results are discussed in the context of the experimentally observed species in Figure 1b–e.

## Computational Details

The QM/MM model was constructed based on the E_0_ model of the MoFe protein described previously.^18^ This model has been shown to accurately reproduce the cofactor geometry of the corresponding 1.0 Å high-resolution X-ray structure. The QM region in this work includes FeMoco, all residues that directly bind FeMoco, as well as proximal charged residues, and residues surrounding Fe2 and Fe6: FeMoco, homocitrate, His442, Cys275, His195, Gln191, Val70, Arg96, Arg359, Tyr229, and Ser278. The protein backbone of the residues was not included, except for the Ser residue, because the amide forms a hydrogen bond with the sulfide of Cys275. The QM region is shown in Figure S1. For the E_1_ QM/MM model, an additional proton was added to the S2B belt sulfide following a recently combined QM/MM and extended X-ray absorption fine structure (EXAFS) study that suggested protonation of a belt sulfide (S2B or possibly S5A) to occur in the E_1_ redox state.^20^ The CO-bound structures reported herein were optimized by relaxing the active region (about 1000 atoms). CO was initially placed at about 1.8 Å from either Fe6 or Fe2. CO and SH^–^ in the E_1_ models were found to coordinate both in a terminal and in a bridging mode depending on the tested broken-symmetry (BS) determinant (*vide infra*). Therefore, all combinations of (i) a terminal CO and a terminal SH^–^, (ii) a terminal CO and a bridging μ-SH^–^, and (iii) a bridging μ-CO and a terminal SH^–^ were tested in order to find all relevant local minima. In the QM/MM ΔHis195 model, the atoms of the His195 side chain were simply deleted from the QM region, and the His195 residue is therefore completely absent in this model. The QM/MM ΔHis195 model is not further optimized after the deletion, and the energies reported refer to the energy of the QM region in the field of the MM charges.

The QM regions were calculated with the ORCA program suite, version 4.2.^55−57^ The hybrid density functional TPSSh was used, which includes 10% Hartree–Fock (HF) exchange.^58,59^ A low HF exchange in the hybrid density functional has been shown to yield accurate geometries for FeS clusters.^60−62^ Furthermore, calculations with TPSSh have successfully reproduced the X-ray structure of the resting state MoFe protein^18,63^ as well as key features in the X-ray absorption spectroscopy (XAS) spectra of FeS clusters, such as the relationship between the pre-edge area in S K-edge XAS spectra and the Fe oxidation state in Fe_2_S_2_ dimers.^64^

Dispersion forces were approximated with the atom-pairwise post-DFT correction by Grimme including Becke–Johnson damping (D3BJ in ORCA).^65,66^ Scalar relativistic effects were modeled by the zeroth order regular approximation (ZORA).^67,68^ All electron basis sets of the Karlsruhe-type recontracted for ZORA were used (ZORA-def2-XVP).^69,70^ Unless noted otherwise, the basis set of triple-ζ quality (X = TZ) was used for all metals, the sulfurs, the central carbide, the CO molecule, the added proton in the E_1_ models, and the homocitrate, while double-ζ (X = S) was used for the remaining atoms. Coulomb and HF exchange integrals were approximated with resolution of identity Coulomb approximation and chain-of-spheres exchange (RIJCOSX)^71,72^ employing the auxiliary basis set SARC/J.^70^

The CHARMM36 force field was used to describe the non-QM protein environment, which was modified to include non-bonding parameters for the FeS clusters as previously described.^18,73^ The coupling between the QM and MM Hamiltonians (using electrostatic embedding) was calculated using a custom version of ChemShell, based on version 3.7.^74,75^ A link to the ChemShell setup including the modified parameter files is made available in the Supporting Information.

The nomenclature of the BS determinants in this paper follows the classification introduced by Noodleman *et al.* with the added three-digit label indicating the spin-flipped Fe centers.^76^ For example, the label BS7-235 indicates that in this BS determinant the centers Fe2, Fe3, and Fe5 carry an excess β spin and that the determinant belongs to the Noodleman class 7. The members of a class are related by the symmetry operations of the *C*_3*v*_ point group. A BS determinant was generated with the FlipSpin procedure implemented in ORCA. The high-spin multiplicity for the resting state, substrate-free cofactor was 36, assuming all local high-spin metal centers and the formal oxidation states [Fe_3_^II^Fe_4_^III^Mo^III^S_9_C]^1–^ (number of unpaired electrons: 3 × 4 (Fe^2+^) + 4 × 5 (Fe^3+^) + 3(Mo^3+^) = 35).^17^ The high-spin multiplicity was reduced by 2 for each E_n_ → E_n+1_ reduction event because the reduction is expected to be Fe-centered (*i.e.* Fe^3+^ → Fe^2+^),^20^ and it is further reduced by 2 when CO was bound because CO has been shown to induce local spin-pairing (*vide infra*). The calculated BS determinants include the three members of the BS7 class (BS7-235, BS7-247, and BS7-346), because they have been shown to constitute the lowest-energy models for the resting state FeMoco.^18,77−79^ Also, the BS10-147 determinant has been calculated because it constitutes a low-energy model for the QM/MM models of the E_4_ state,^63,80^ as well as BS10-135, which is the mirror image of BS10-147 with respect to Fe6/Fe2.

The cluster models were created from the substrate-free E_0_ QM/MM model (BS7-235 with *M*_S_ = 3/2).^18^ The cluster models consist only of the cofactor, the homocitrate, the His residue bound to Mo, and the Cys residue bound to Fe1. The computational protocol was analogous to the QM/MM models with three exceptions: (1) the triple-ζ basis was used for all atoms. (2) Instead of the MM embedding, the surroundings were described with the conductor-like polarizable continuum model (C-PCM) with a dielectric constant of ϵ = 4.^81,82^ (3) During the geometry optimizations, the position of the homocitrate, His, and Cys residue were constrained; only the cofactor [Fe_7_MoS_9_C] and CO were allowed to relax.

CO binding energies were calculated as Δ*E* = *E*_AB_ – (*E*_A_ + *E*_B_), where *E*_AB_, *E*_A_, and *E*_B_ are the total electronic energies of the CO-bound system, the substrate-free system, and unbound CO, respectively. The electronic energies correspond reasonably well to the enthalpy of the cofactor. The calculated binding enthalpy Δ*H* differs by less than 1 kcal/mol from the binding electronic energy (tested for the E_1_ cluster model with CO bound to Fe6 considering all vibrations of CO and FeMoco: Δ*E* = −15.0 kcal/mol and Δ*H* = −14.1 kcal/mol). Entropic effects are discussed separately in the text.

The total electronic energies of all tested substrate-free models are shown in Figure S3, and those of all tested CO-bound models are given in Figure S4 (cluster model) and Figure S5 (QM/MM model). The metal–metal distances for the lowest-energy models are given in Table S2, and the geometries of the respective QM regions are attached as xyz files.

The CO vibrational frequency was calculated by diagonalizing the partial Hessian matrix of only the CO coordinates and the binding Fe center(s). It was found that the frequency remains virtually the same when including more atoms (see Table S3). All reported frequencies were scaled so that the as-calculated frequency of CO in vacuum (2181 cm^–1^) matches the experimental IR frequency of CO gas (2143 cm^–1^).^83^ Therefore, all calculated frequencies were multiplied by 0.9826. Both as-calculated and scaled frequencies are listed in Table S3.

In diamagnetic substitution, most magnetic metal centers are replaced by diamagnetic ions with a similar ionic radius, which has proven useful for studying the complex electronic structure of FeS clusters.^84−86^ According to the localized orbital analysis of the complete cofactors, the Fe centers in FeMoco have an intermediate oxidation state in between Fe^3+^ and Fe^2+^ (a result of delocalized electrons being shared between Fe centers^18^) and neither Ga^3+^ nor Zn^2+^ are a suitable diamagnetic equivalent for Fe in FeMoco. To emulate an intermediate oxidation state, the atomic charge of Ga was lowered to *Z* = 30.5, as this resulted in sulfide atomic charges closer to the unsubstituted cofactors compared to normal Ga atoms (*Z* = 31) or normal Zn atoms (*Z* = 30). Therefore, diamagnetically substituted cofactors were constructed by replacing Mo with In and Fe with modified Ga (*Z* = 30.5). For the CO–[FeGa_6_InS_9_C]^2+,1+,0^ and CO–[Fe_2_Ga_5_InS_9_C]^1+^ models, the lowest-energy E_0_ cluster model with CO bound to Fe6 was used and all metal centers but Fe6 (or Fe6/Fe2) were substituted. Only the positions of CO were relaxed to account for CO activation and in order to calculate the CO frequency. Analogous calculations for CO bound to Fe2 led to qualitatively equivalent results. For μ-CO–[Fe_2_Ga_5_InHS_9_C]^2+^, the structure of the converged E_1_ QM/MM model with CO bridging Fe6 and Fe2 was used (see Figure 5). The CO coordinates were not optimized further after the diamagnetic substitution because the bridging binding motif was only stable for the E_1_ QM/MM model. Therefore, no CO frequency was calculated for μ-CO–[Fe_2_Ga_5_InHS_9_C].

Localized orbitals were generated with the Foster–Boys algorithm as implemented in ORCA.^87^ The complete set of metal/CO-based localized orbitals is given for the QM/MM models in Figure S6 (substrate-free), Figure S7 (CO-bound in E_0_), and Figure S8 (CO-bound in E_1_). All orbital populations, atomic charges, and spin populations are reported within the Hirshfeld partitioning scheme,^88^ as implemented in the wave function analyzer program Multiwfn,^89^ version 3.7, using precalculated neutral atom densities to construct the density of the promolecule (default in Multiwfn). The Hirshfeld-I scheme was also tested, which is an extension to the original scheme, where the atomic volumes and hence the charges are iteratively refined^90^ but was found unstable as the charge of the central carbide diverges. The atomic spin populations and charges of the lowest-energy models are listed in Tables S5 and S6, respectively. Molecules and orbitals were rendered with the VMD visualization program, version 1.9.3.^91^ Orbitals are plotted with isosurfaces at ±0.05 (solid) and ±0.025 (transparent). Atom colors are Mo: purple, Fe: green, Ga/In: black, S: yellow, C: gray, O: red, N: blue, H: white.

## Results and Discussion

### Electronic Structure of FeCO Fragments

The electronic structure of a transition-metal complex depends on its ligand field, which is determined by the coordination geometry and the nature of the ligands.^92^ CO induces a large ligand field splitting and typically leads to spin-pairing at the metal site.^93^ π back bonding from suitable metal orbitals to the antibonding π* orbitals on CO weakens and therefore activates the C–O bond. On the other hand, the tetrahedral Fe sites in biological FeS clusters usually maintain a local high spin in the weak ligand field of the S^2–^ ligands. It is not *a priori* evident how the interaction between CO and the Fe centers in FeMoco relates to these two extremes, and it is therefore instructive to discuss the influence of a CO ligand on the local electronic structure of an Fe center in the chemical environment of FeMoco. To this end, all metal centers except the center(s) of interest were replaced by a diamagnetic metal ion in a simple cluster model of the cofactor: Ga^3+^ replaces Fe and In^3+^ replaces Mo, both of which have a d^10^ configuration. These ions have similar ionic radii as Fe and Mo, respectively, in the chemical environment of FeMoco and therefore exert a similar electrostatic influence, but they do not engage in the more complicated spin–spin interaction. Thus, diamagnetic substitution generates Fe–CO fragments that are more relevant for a CO–FeMoco adduct than, for example, model complexes with multiple CO ligands coordinated to the same Fe center. This partial substitution of magnetic centers with diamagnetic ions has been shown to aid the understanding of the complex electronic structure in FeS clusters by separating local and cooperative effects.^84−86^

#### Terminal CO Binding

**Figure 2:**
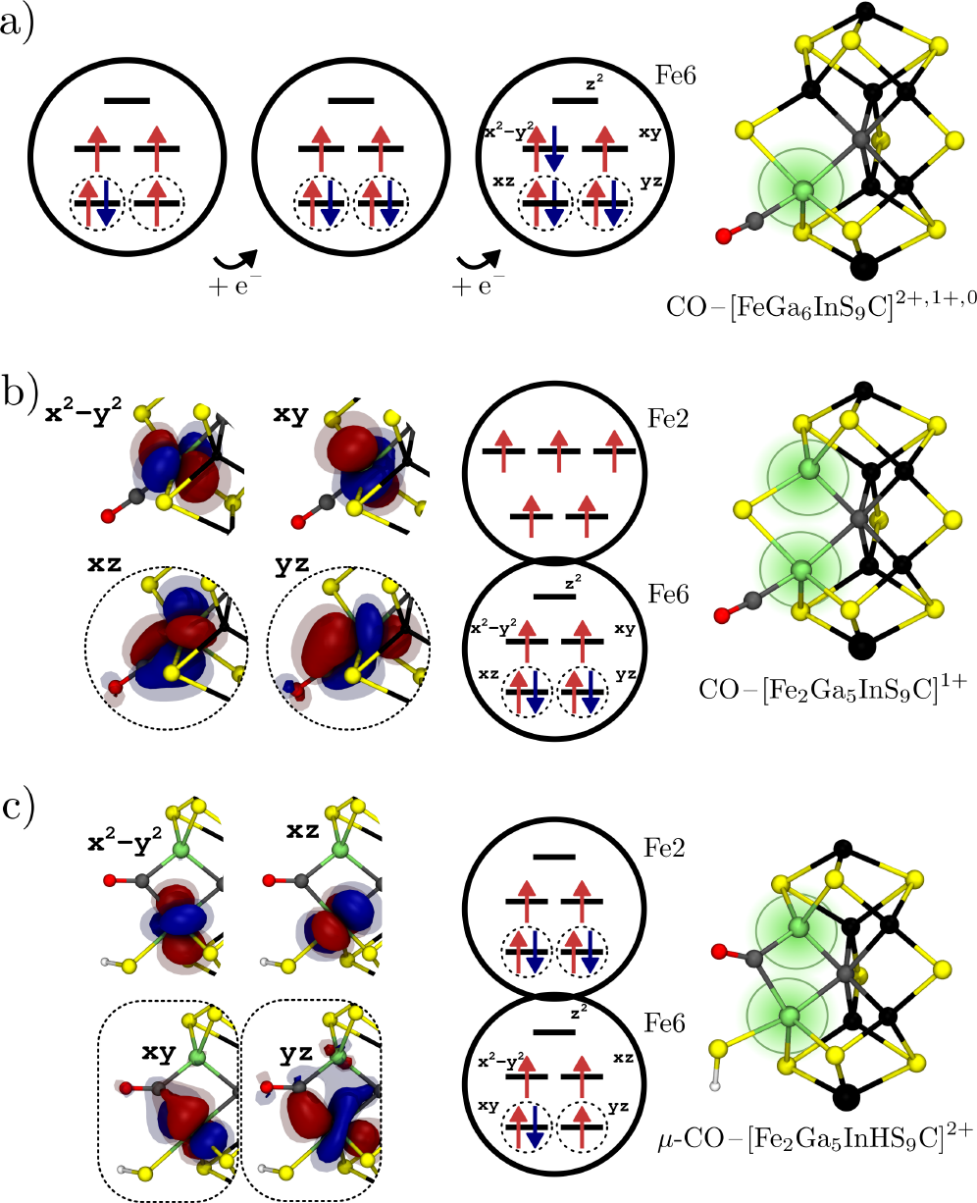
Structures of the diamagnetically substituted CO-bound cofactors and the localized orbitals for the indicated charge states: (a) CO bound terminally to the single Fe center in [FeGa_6_InS_9_C]^2+,1+,0^. (b) CO bound terminally to one of two Fe centers in [Fe_2_Ga_5_InS_9_C]^1+^. (c) CO bridging the two Fe centers in [Fe_2_Ga_5_InHS_9_C]^2+^ (open SH^–^ bridge). Diamagnetic metal centers are shown in black (Fe/Mo replaced with Ga/In, respectively). Orbitals with significant Fe–CO overlap are highlighted by dashed circles. The orbital labels correspond to a local coordinate system in which the *z* axis is oriented along the respective Fe–carbide bond and the *x* and the *y* axes lie within and perpendicular to the Fe2/Fe6/carbide plane, respectively. For orbitals that are not shown: the Fe2 orbitals (b,c) are mirror images of Fe6. The Fe6 orbitals in (a) are equivalent to the Fe6 orbitals in (b).

The simplest and the most common CO binding mode is a terminal coordination of a metal center *via* the carbon atom. To study this binding mode in FeMoco, we created an [FeGa_6_InS_9_C] complex by substituting all Fe except the Fe6 center by Ga and furthermore Mo by In. These diamagnetic substituents are shown with black atoms in Figure 2a. Similarly, binding CO to Fe2 in the Fe-only cubane was found to give qualitatively equivalent results.

Prior to CO binding, the single Fe center in [FeGa_6_InS_9_C]^2+,1+,0^ is a high-spin center for the three oxidation states Fe^3+,2+,1+^. This is in line with the expectations for an approximately tetrahedral, weak ligand field created by three sulfides and one carbide ligand. Binding CO leads to stable, bound structures with a pentacoordinate Fe center for all three Fe oxidation states (CO–[FeGa_6_InS_9_C]^2+,1+,0^). In all three cases, an intermediate spin state is the most stable, as can be seen in the corresponding orbital occupation schemes in Figure 2a. The *z*^2^ orbital is exclusively unoccupied. Going from oxidized to more reduced Fe, the orbitals are filled in the order *xz*, *yz*, and *x*^2^–*y*^2^. This behavior meets the expectations for a CO ligand, which is both a σ-donor and a π-acceptor and therefore destabilizes Fe orbitals along the Fe–CO bond (*z*^2^) but stabilizes the Fe orbitals that overlap with the CO π* orbitals (*xz* and *yz*).

The Fe–CO distance in CO–[FeGa_6_InS_9_C]^2+,1+,0^ decreases from 1.85 to 1.76 to 1.71 Å, respectively (see Table 1). At the same time, the C–O bond length is 0.017, 0.032, and 0.054 Å longer compared to unbound CO (1.129 Å at the same level of theory). The shorter Fe–CO and the longer C–O bond correlate with the more electron-rich Fe center in [FeGa_6_InS_9_C]. Note that the change in the C–O bond length is larger between Fe^3+^ and Fe^2+^, but the change in the Fe–CO bond length is larger between Fe^2+^ and Fe^1+^. Therefore, the amount of CO activation does not necessarily correlate with the length of the Fe–CO bond.

**Table 1:** Fe–CO Bond Parameters in the Diamagnetically Substituted Cofactors That Relate to CO Activation^a^

		*S*_pop_^b^				
	binding mode	Fe6	Fe2	ν_CO_^c^ [cm^–1^]	*d*_C–O_ [Å]	*d*_Fe–CO_ [Å]
CO–[FeGa_6_InS_9_C]^2+,1+,0^	CO–Fe^3+^	2.50		1978	1.146	1.850
	CO–Fe^2+^	1.84		1884	1.161	1.756
	CO–Fe^1+^	1.04		1772	1.183	1.710
CO–[Fe_2_Ga_5_InS_9_C]^1+^	CO–Fe^2+^Fe^3+^	2.05	3.58	1908	1.157	1.768
μ-CO–[Fe_2_Ga_5_InHS_9_C]^2+^	μ-CO–Fe^2+^Fe^3+^	2.29	2.50		1.193	1.837/1.960

a Corresponding structures and orbital occupation schemes are shown in Figure 2.

b Spin populations are based on the Hirshfeld partitioning scheme.

c Scaled vibrational frequencies. See Table S3 for unscaled frequencies.

Similar to the C–O bond length, the calculated CO vibrational frequency in CO–[FeGa_6_InS_9_C]^2+,1+,0^ captures CO activation and decreases from 1978 to 1884 to 1772 cm^–1^, respectively. The frequencies for the Fe^3+^ and Fe^2+^ oxidation states correspond reasonably well to experimentally observed frequencies for terminally bound CO to FeMoco (between 1900 and 1980 cm^–1^).^37−39,42^ The calculated CO frequency is lowered by about 100 cm^–1^ upon the reduction of Fe^3+→2+^ as well as Fe^2+→1+^. The additional electron in the Fe^1+^ oxidation state occupies the *x*^2^–*y*^2^ orbital (no overlap with CO π* orbitals), but the overlap of the *xz* and *yz* orbitals with CO increases for Fe^2+→1+^. Therefore, reducing an Fe center also activates the CO bond even if the additional electron is not directly involved in the π back bond.

Next, the influence of a neighboring Fe center on the Fe–CO bond is explored by “reactivating” Fe2 in addition to Fe6 while keeping diamagnetic ions for the remaining metal centers (CO–[Fe_2_Ga_5_InS_9_C]^1+^ in Figure 2b). According to BS-DFT models of the resting state FeMoco, both Fe6 and Fe2 have Fe^2.5+^ oxidation states due to being part of delocalized mixed-valence pairs in their respective cubanes.^18^ CO binding to Fe6 leads to charge localization, and the oxidation states of Fe6 and Fe2 become Fe^2+^ and Fe^3+^, respectively. The orbital occupation pattern on Fe6 is analogous to Fe^2+^ in CO–[FeGa_6_InS_9_C]^1+^, with an unoccupied *z*^2^ orbital and doubly occupied *xz* and *yz* orbitals resulting in the same local intermediate spin. The neighboring Fe2 has a local highspin.

Compared to CO–[FeGa_6_InS_9_C]^1+^, the Fe–CO bond length in is 0.012 Å longer, the C–O bond length is 0.004 Å shorter, and the CO frequency is 24 cm^–1^ higher (see Table 1). Therefore, the strength of the Fe–CO bond and the amount of CO activation is slightly lower in (Fe^2+^Fe^3+^ fragment) compared to CO–[FeGa_6_InS_9_C]^1+^ (Fe^2+^ fragment). A closer inspection of the orbitals in CO–[Fe_2_Ga_5_InS_9_C]^1+^ reveals that some Fe6-based localized orbitals also show small contributions from Fe2. Because the neighboring Fe center allows for electron delocalization toward Fe2, the Fe–CO bond is weakened compared to a single Fe^2+^ magnetic center.

In biological [Fe_2_S_2_]^2+^ clusters, the two local high-spin ferric centers typically exhibit a strong antiferromagnetic coupling to a diamagnetic ground state.^94^ Thinking of larger FeS clusters as consisting of smaller fragments, it has been proposed for FeMoco that those spin alignments, that is, BS determinants, are the most stable that contain the maximum number of antiferromagnetically coupled Fe pairs.^76,95^ These are the determinants belonging to the BS7 class, namely, BS7-235, BS7-247, and BS7-346, and they constitute the lowest-energy determinants in the resting state MoFe QM/MM model.^18,77−79^ The coupling strength between two magnetic centers can be quantified by the coupling constant *J*. Using the spin Hamiltonian *Ĥ*_S_ = −2*JŜ*_A_*Ŝ*_B_, a negative value indicates antiferromagnetic coupling. The coupling constant for an Fe pair can be easily extracted from diamagnetically substituted FeMoco with BS-DFT by keeping only two magnetic centers ([Fe_2_Ga_5_InS_9_C]^1+^). Focusing on the Fe6/Fe2 pair, the calculated coupling constant prior to CO binding is −117 cm^–1^ in [Fe_2_Ga_5_InS_9_C]^1+^, and the two Fe centers are therefore antiferromagnetically coupled. However, after CO binding, the coupling constant for the Fe6/Fe2 pair becomes +34 cm^–1^. The change in the coupling constant with CO binding is clearly a result of the spin-pairing on Fe6 (see Figure 2b), and the local intermediate spin Fe center has a reduced preference for antiferromagnetic alignment with its neighbors. This suggests that the binding of a π-accepting ligand (CO or even N_2_) may affect the stability of BS determinants in FeMoco.

#### Bridging CO Binding

CO bridging Fe6 and Fe2 has been observed in the X-ray structure loCO_XRD_ and has also been proposed as the binding mode in the EPR species loCO_EPR_ and the IR species loCO_IR,2_. We will explain the origins of this bridging CO structure later in this study when discussing the E_1_ QM/MM model but use the structure already at this point to explore the bridging CO binding motif through diamagnetic substitution. In this structure, CO has replaced the S2B belt sulfide as the bridging ligand between Fe6 and Fe2, while the sulfide is still bound to Fe6 as a terminal SH^–^ ligand. The C–O bond in the bridging CO structure is 0.032 Å longer than the terminal CO in CO–[Fe_2_Ga_5_InS_9_C]^1+^ (Table 1). At the same time, the two Fe–CO bonds are about 0.1–0.2 Å longer than the single bond in CO–[Fe_2_Ga_5_InS_9_C]^1+^.

The corresponding diamagnetically substituted model μ-CO–[Fe_2_Ga_5_InHS_9_C]^2+^ is shown in Figure 2c. The orbital occupation scheme indicates the oxidation states Fe^3+^ and Fe^2+^ for Fe6 and Fe2, respectively, which is the opposite electron distribution compared to the terminal CO–[Fe_2_Ga_5_InS_9_C]^1+^. Since CO forms an asymmetric bridge, the reason for the more oxidized Fe6 appears to be the SH^–^ ligand coordinated to Fe6. In contrast to the substrate-free FeMoco, both Fe6 and Fe2 exhibit a local intermediate spin state because the *z*^2^ orbital, which is oriented along the respective Fe–carbide bond, is unoccupied in both centers. This definition of the local coordinate systems on Fe6 and Fe2 also implies that the Fe orbitals which overlap with the π* orbitals of the bridging CO are the *xy* and the *yz* orbitals (in contrast to the *xz* and *yz* orbitals for terminal CO). Furthermore, one has to distinguish between the in-plane and out-of-plane contributions to the π back bonding. The *xy* orbital (out-of-plane) is doubly occupied on both Fe centers, but the *yz* orbital (in-plane) is doubly occupied only on Fe2. Therefore, the out-of-plane π back bonding *via* the *xy* orbitals appears to be energetically favored for a bridging CO in FeMoco. The longer C–O bond length compared to terminal CO, despite the significantly longer Fe–CO bond lengths, is consistent with two Fe centers contributing to the π back bonding instead of one.

The calculated coupling constant for the Fe6/Fe2 pair in μ-CO–[Fe_2_Ga_5_InHS_9_C]^2+^ is +47 cm^–1^. The ferromagnetic coupling of Fe6 and Fe2 bridged by CO is even stronger compared to the terminally bound CO in CO–[Fe_2_Ga_5_InS_9_C]^1+^ (+34 cm^–1^). The reason could be either the additional spin-pairing or the replacement of the bridging μ-S^2–^, which favors antiferromagnetic coupling in Fe_2_S_2_ dimers.^85^

#### Lessons Learned from Diamagnetic Substitution

The study of Fe–CO fragments in diamagnetically substituted FeMoco allows for a simplified correlation between CO activation and the local electronic structure of an Fe center in the chemical environment of FeMoco. The different oxidation states in CO–[FeGa_6_InS_9_C]^2+,1+,0^ show a clear correspondence between the Fe electron configuration and CO activation (Figure 2a). A more reduced Fe center leads to a lower CO vibrational frequency, a shorter CO bond length, and a longer Fe–CO bond length. The CO frequency for an Fe^2+^ oxidation state (1884 cm^–1^, CO–[FeGa_6_InS_9_C]^1+^) is the closest to the experimental frequency for the putative terminal CO observed experimentally in FeMoco by SF-FT-IR spectroscopy (1904 cm^–1^, loCO_IR,1_). An intermediate spin state is preferred for all three oxidation states after CO binding, which is in between the local high-spin state typically observed in most biological, substrate-free FeS clusters and the local low-spin state for multiple CO bound to the same Fe center.^93^ The localized orbital analysis reveals that local spin-pairing increases π back bonding, which is responsible for the CO activation.

The CO–[Fe_2_Ga_5_InS_9_C]^1+^ model shows that the second center has only a small effect on the CO activation compared to CO–[FeGa_6_InS_9_C]^1+^ (Figure 2a,b). However, the local spin-pairing on the CO-bound Fe center changes the sign of the coupling constant between the neighboring Fe centers. The μ-CO binding mode in μ-CO–[Fe_2_Ga_5_InHS_9_C]^2+^ (Figure 2c) shows even stronger ferromagnetic coupling between Fe6 and Fe2 compared to terminal CO binding. The stability of a particular spin-coupling pattern within FeMoco is believed to depend greatly on antiferromagnetic coupling between the Fe centers.^76,95^ CO binding might thus be expected to disrupt the energy ordering of BS determinants through local spin-pairing and by potentially replacing the bridging μ-S^2–^ ligand.

### CO Binding to the E_0_ State of FeMoco

We now turn to the full FeMoco model in the E_0_ state and consider the interaction between all eight magnetic centers. However, we will restrict our study to CO binding to Fe6 and Fe2 as X-ray crystallography has revealed CO binding to these atoms in the X-ray structure loCO_XRD_. The first part of this section presents the results for a cluster model. In this model, only the cofactor and the residues with direct coordination to Fe1 and Mo are included. The remaining protein environment is replaced with a uniform dielectric continuum (ϵ = 4). Even though CO binding to the wild-type MoFe protein generally requires turnover conditions, we consider it important to understand how an Fe–CO bond affects the spin-coupling in the full FeMoco model and the E_0_ state is by far the best understood E_n_ state. The second part shows analogous calculations with a more realistic QM/MM model. The residues in the proximity of Fe6 and Fe2 are included in the QM region, that is, modeled with DFT, while the remaining residues are modeled with a molecular mechanics force field. By comparing the cluster model with the QM/MM model, the effects of the surrounding residues on the CO–FeMoco adduct can be discussed separately from the intrinsic electronic structure of FeMoco.

#### CO Binding in a Cluster Model

**Figure 3:**
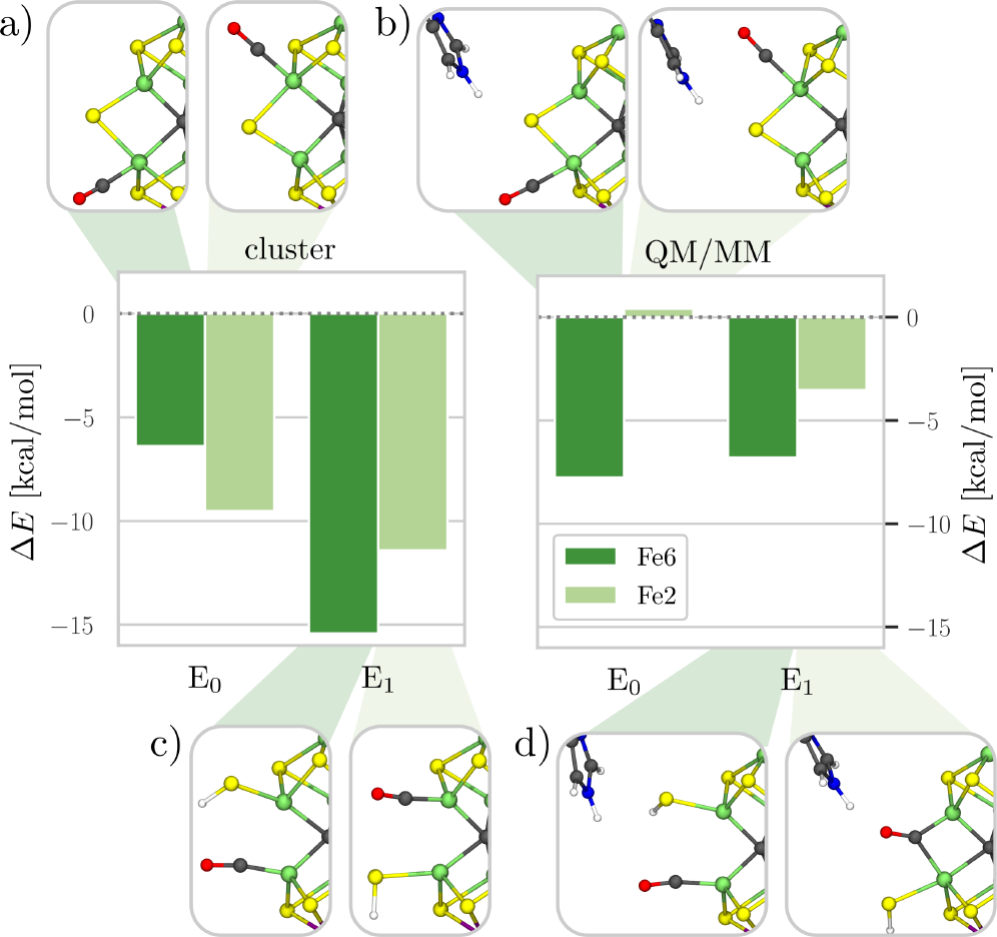
CO binding modes and binding energies for the cluster model (a,c) and the QM/MM model (b,d) for both the E_0_ and E_1_ redox states, respectively. The QM/MM model explicitly includes the protein environment such as the His195 residue. For the E_1_ state, the protonated S2B belt sulfide bridge opens spontaneously. When binding CO to Fe2 in the E_1_ QM/MM model, CO assumes the bridging position between Fe6 and Fe2. Cf. Figure S3 for substrate-free reference states and Figures S4 and S5 for other BS determinants tested.

The E_0_ cluster model converges to stable structures with CO bound terminally to either Fe6 or Fe2, as illustrated in Figure 3a. The binding energies are calculated to be −6.0 and −9.1 kcal/mol, respectively, which shows a clear preference for CO binding to Fe2. The remaining parameters related to the Fe–CO bond only differ slightly between the two binding sites (see Table 2). The calculated vibrational frequencies are 1910 and 1896 cm^–1^ for CO bound to Fe6 and Fe2, respectively. This is in good agreement with the CO bound terminally to the diamagnetically substituted cofactor CO–[Fe_2_Ga_5_InS_9_C]^1+^ (1908 cm^–1^). The lower vibrational frequency for CO bound to Fe2 (−14 cm^–1^) correlates with a slightly shorter Fe–CO bond length (−0.04 Å) compared to Fe6, while the C–O bond length is nearly unchanged (+0.001 Å).

**Table 2:** Parameters That Characterize the CO–FeMoco Interaction in the Cluster Model and in the QM/MM Model^a^

		|*S*_pop_|^b^							
		Fe6	Fe2	Δ*E*^c^ [kcal/mol]	ν_CO_^d^ [cm–1]	*d*_C–O_ [Å]	*d*_Fe–CO_ [Å]	BS^e^	*M*_S_^f^
E_0_ models									
cluster	Fe6	1.56	3.19	–6.0	1910	1.158	1.760	BS10-135	0.5
	Fe2	2.93	1.95	–9.1	1896	1.159	1.756	BS10-147	0.5
QM/MM	Fe6	1.51	3.06	–7.8	1966	1.152	1.763	BS10-147	0.5
	Fe2	2.96	1.81	0.4	1957	1.153	1.775	BS7-346	0.5
E_1_ models									
cluster	Fe6	1.07	3.22	–15.0	1821	1.171	1.735	BS10-147	0.0
	Fe2	3.00	1.61	–11.0	1856	1.166	1.745	BS10-147	0.0
QM/MM	Fe6	1.88	3.16	–8.3	1922	1.157	1.783	BS7-346	2.0
	Fe6/2	2.26	2.11	–5.1	1716	1.193	2.022/1.795	BS10-147	0.0

a The respective binding modes are shown in Figure 3 (all models), Figure 4 (E_0_ QM/MM), and Figure 5 (E_1_ QM/MM).

b Spin populations are based on the Hirshfeld partitioning scheme.

c The calculation of the binding energy Δ*E* is explained in the Computational Details section.

d Scaled vibrational frequencies. See Table S3 for unscaled frequencies.

e BS refers to the spin-coupling within the broken symmetry determinant (most stable shown), with the last three integers indicating the Fe centers that hold mainly β spin electrons.

f *M*_S_ refers to the excess of α spin in the BS determinant.

Before CO binding, Fe6 (or Fe2) is a local high-spin center with an Fe^2.5+^ oxidation state, because it shares a minority spin electron with a neighboring metal center.^18^ The reader is referred to Figure S2 for a schematic electronic structure of the whole, substrate-free cofactor. When binding CO, the shared electron localizes, forming an Fe^2+^Fe^3+^ pair, where Fe6 (or Fe2) is the more reduced Fe center. Fe6 (or Fe2) has a local intermediate spin, with an unoccupied *z*^2^ orbital (oriented along the Fe–CO bond) and doubly occupied *xz* and *yz* orbitals (constituting the π back bonding). Therefore, the orbital diagram of CO–[Fe_2_Ga_5_InS_9_C]^1+^ (Figure 2b) captures the electron reorganization that happens when binding CO with the difference that the Fe^2+^/Fe^3+^ pair is located within the respective cubane (Fe6/Fe5 and the Fe2/Fe4 pair, respectively).

In addition to the spin-pairing, the global spin-coupling pattern in FeMoco changes upon CO binding and with it the location of the mixed-valence delocalized pairs. In the substrate-free FeMoco, the members of the BS7 class (BS7-235, BS7-247, and BS7-346) have been shown to be the lowest in energy,^18,77−79^ which has been rationalized through the high number of antiferromagnetically aligned Fe pairs.^76,95^ However, the study of the diamagnetically substituted cofactor showed that a terminally bound CO reduces the coupling constant between Fe centers by inducing local spin-pairing. Because the same local intermediate spin is observed for the CO-bound Fe center in the full FeMoco models, the local intermediate spin center might contribute less to the stability of the BS determinant through an antiferromagnetic alignment compared to the remaining Fe pairs. The most stable determinants after CO binding are BS10-135 and BS10-147 for CO bound to Fe6 and Fe2, respectively. BS10-135 and BS10-147 are pseudo-mirror images with respect to the plane defined by Fe6, Fe2, and CO. If the local intermediate spin center is simply disregarded when counting the number of antiferromagnetic pairs, the BS7 class loses its leading position and the BS2, BS4, BS6, BS7, BS8, and BS10 classes all have the same, maximum number of antiferromagnetic pairs. The reduced coupling strengths of the CO-bound Fe center offers an intuitive explanation as to why the lowest-energy BS determinants do not necessarily belong to the BS7 class. It further reinforces the importance of understanding the relationship between the electronic structure and the stability of BS determinants in the ligand-bound FeMoco.

The electronic structure of FeMoco in the E_0_ cluster model changes in a similar way when binding CO to Fe6, which is part of the Mo cubane, and to Fe2, which is part of the Fe-only cubane. However, the binding energies show a preference by about 3 kcal/mol for binding to Fe2. Because the two binding sites do not show any significant difference in the CO vibrational frequency or geometric parameters, the preference for Fe2 is related to the electronic structure of the respective cubanes. It appears that altering the electronic structure through CO binding, that is, localization of a formerly delocalized electron and/or local spin-pairing, happens more easily in the Fe-only cubane compared to the Mo cubane. This observation is possibly a consequence of the heterometal Mo, and the unusually strong Fe–Mo interaction in FeMoco^96^ could play a role in stabilizing local high-spin Fe centers.

#### CO Binding in a QM/MM Model

In the E_0_ QM/MM model, placing CO in the proximity of either Fe6 or Fe2 converges to a structure with a terminally bound CO, similar to the cluster model, as shown in Figure 3b. The binding energies are −7.8 and 0.4 kcal/mol for Fe6 and Fe2, respectively. Compared to the cluster model, CO binding is only 2 kcal/mol weaker for Fe6, but 10 kcal/mol weaker for Fe2, which indicates a strong influence of the protein environment. Indeed, the overlay of the CO-bound and substrate-free structures shows a large displacement of His195 if CO binds to Fe2 but a small displacement for Fe6 (Figure 4a,b). In order to verify the influence of the His residue, the binding energies were recalculated with the His residue removed from the QM/MM model at a fixed geometry (QM/MM ΔHis195 model). Figure 4c compares the binding energy difference, that is, the preference of CO to bind to either Fe6 or Fe2, between the cluster model, the QM/MM model, and the QM/MM ΔHis195 model. A positive value for the binding energy difference indicates a preference for Fe2. The QM/MM model has a clear preference for binding at Fe6 (−8.2 kcal/mol), but the preference for Fe2 in the QM/MM ΔHis195 model (+3.1 kcal/mol) is nearly identical to the cluster model (+3.8 kcal/mol), demonstrating the role of the His residue. The His residue forms a hydrogen bond with the belt sulfide S2B, and this bond competes with the substrate binding to Fe. The increase of the binding energy difference by 10 kcal/mol makes Fe2 an unfavorable binding site in the E_0_ QM/MM model, where the His–S2B hydrogen bond is still intact. This emphasizes the important role the protein environment can play in mechanistic studies of nitrogenase.

**Figure 4:**
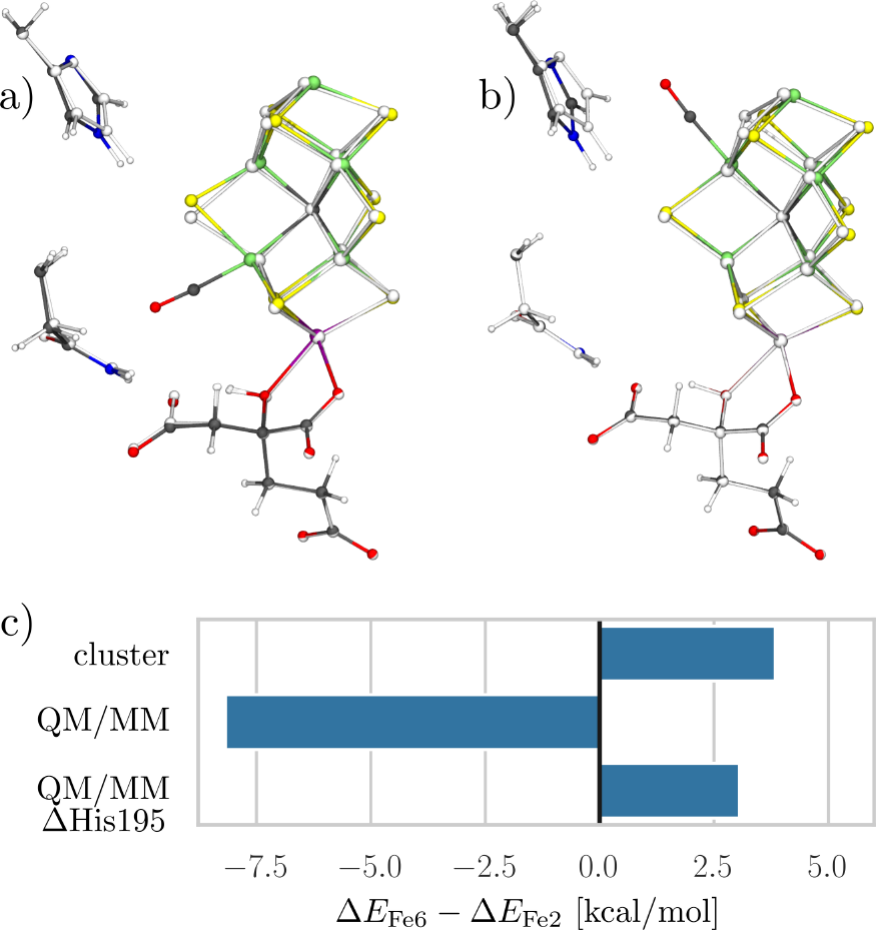
Structures of CO bound in the E_0_ QM/MM model to Fe6 (a) and Fe2 (b) showing also the His195 and Gln191 residues. The substrate-free model is overlaid in black and white. (c) Energy difference between the CO binding at Fe6 and at Fe2 in the cluster model, the QM/MM model, and the QM/MM model with His195 removed at a fixed geometry (ΔHis195). Positive values indicate a preference for binding to Fe2.

The vibrational frequency is very similar for CO bound to Fe6 (1966 cm^–1^) and to Fe2 (1957 cm^–1^). While the vibrational frequency is nearly unaffected by the 8 kcal/mol difference in binding energy between Fe6 and Fe2, the stronger binding to Fe6 is accompanied by a 0.01 Å decreased Fe–CO bond length compared to Fe2. The frequency for a supposedly terminal CO observed in SF-FT-IR experiments (1904 cm^–1^, loCO_IR,1_)^38,39^ coincides with the calculated frequencies for the E_0_ cluster model (1910 and 1896 cm^–1^ for Fe6 and Fe2, respectively), but it is about 50 cm^–1^ lower than the calculated frequencies for the E_0_ QM/MM model (1966 and 1957 cm^–1^ for Fe6 and Fe2, respectively). The discrepancy in the frequency between the cluster and the QM/MM model is a direct consequence of the respective descriptions of the protein environment.

The localized orbital analysis of CO-bound FeMoco in the E_0_ QM/MM model revealed the equivalent orbital occupation scheme as discussed for the E_0_ cluster model. To reiterate, the CO binding leads to the localization of a formerly delocalized electron and therefore a formal Fe^2+^Fe^3+^ pair with a local intermediate spin on the CO-bound Fe^2+^ center. The lowest-energy determinant is BS10-147 and BS7-346 for CO bound to Fe6 and Fe2, respectively. BS7-346 is not the lowest-energy determinant in the cluster model, but as already discussed, the BS7 and BS10 classes have the same number of antiferromagnetically coupled Fe pairs when neglecting the local intermediate spin Fe center. The observation that the lowest-energy BS determinant differs between the cluster and the QM/MM model demonstrates that the explicit protein environment has a considerable influence on stabilizing certain spin-coupling patterns.

CO binding to FeMoco in the wild-type MoFe protein generally requires turnover conditions. Consistent with this observation, the calculated CO frequencies in the E_0_ QM/MM model (1966 and 1957 cm^–1^ for Fe6 an Fe2, respectively) are about 50 cm^–1^ higher than the initial, experimental band at 1904 cm^–1^ in the SF-FT-IR experiment (loCO_IR,1_), which suggests that a more reduced cofactor is present in the experiment. Arguably, the discrepancy is not sufficiently large to exclude the E_0_ redox state as the initial binding state based on the frequency alone. Nevertheless, the binding energy difference between Fe6 and Fe2 shows that ligand binding to Fe2 is strongly hindered as long as the His195 residue forms a hydrogen bond with the belt sulfide S2B.

### CO Binding to the E_1_ State of FeMoco

We now turn our attention to the one-electron reduced and protonated E_1_ state. It has been proposed that the additional electron reduces the Fe part of the Mo cubane,^20,21^ which is therefore expected to affect the Fe–CO interaction. The S2B belt sulfide is protonated in our model following the suggestion of a combined EXAFS and QM/MM study, but we note that the S5A position has also been proposed as a protonation site.^20,22−25^ Similar to the E_0_ models, the first part of this section is dedicated to intrinsic properties of FeMoco and investigates CO binding using the cluster model. The second part focuses on the influence of the protein environment on the Fe–CO bond by repeating the calculations with the QM/MM model.

#### CO Binding in a Cluster Model

The E_1_ cluster model binds CO terminally at Fe6 or Fe2, as shown in Figure 3c. The binding of CO is accompanied by the spontaneous opening of the bridge formed by the protonated S2B belt sulfide between Fe6 and Fe2, resulting in a terminal CO on one Fe center and a terminal SH^–^ on the other. The binding energies are −15.0 and −11.0 kcal/mol for CO bound to Fe6 and Fe2, respectively, which makes the binding 9 and 2 kcal/mol stronger compared to the E_0_ cluster model for each respective binding site. The stronger CO binding in the E_1_ cluster model is not surprising in view of the more reduced cofactor, but it might also be related to the opening of the SH^–^ bridge, reducing the coordination number at the CO-bound Fe center. CO binding is stronger for Fe6 than for Fe2, which is opposite to the preference observed in the E_0_ cluster model. The localized orbital analysis of the CO-bound E_1_ cluster model (not shown) reveals that the reduction from the E_0_ to the E_1_ redox state occurs in the respective cubane that binds CO (Fe2: Fe-only cubane; Fe6: Mo cubane). The preference for Fe6 is therefore consistent with the electronic structure of the substrate-free E_1_ QM/MM model, which predicts the reduction occurring in the Mo cubane rather than in the Fe-only cubane.^20^

The calculated CO vibrational frequencies in the E_1_ cluster model are 1821 and 1856 cm^–1^ for Fe6 and Fe2, respectively (see Table 2). The 35 cm^–1^ lower frequency for Fe6 correlates with the 4 kcal/mol stronger binding. At the same time, the C–O bond length is 0.005 Å longer for Fe6 and the Fe–CO bond length is −0.010 Å shorter. Compared to the E_0_ cluster model, the CO frequencies decrease by 90 and 40 cm^–1^ for Fe6 and Fe2, respectively, and the Fe–CO bond lengths decrease by 0.025 and 0.010 Å, respectively, revealing a significantly more activated and more strongly bound CO in the cluster model after reduction and protonation to the E_1_ state.

For solution-extracted FeMoco, CO has been shown to not interact with an oxidation state that gives rise to an *S* = 3/2 EPR signal and therefore most likely corresponds to the E_0_ redox state of MoFe-bound FeMoco.^44^ However, solvated FeMoco was found to bind CO after a one-electron electrochemical reduction, where the CO–FeMoco adduct was identified by an IR band at 1835 cm^–1^. Consistent with this observation, our cluster model suggests a stronger binding in the E_1_ state (−15.0 to –11.0 kcal/mol) compared to the E_0_ state (−9.1 to –6.0 kcal/mol). Furthermore, our calculated CO frequencies for the E_1_ cluster model (1821 and 1856 cm^–1^) are in good agreement with the IR band observed at 1835 cm^–1^. While the authors proposed a bridging CO, our model suggests that a terminally bound CO (with an open sulfide bridge) is also consistent with this relatively low IR frequency.

#### CO Binding in a QM/MM Model

The E_1_ QM/MM model predicts two distinct CO binding modes, which are shown in Figure 3d, depending on the initial binding site. When binding CO to Fe6, the CO remains as a terminal ligand on Fe6, but the protonated belt sulfide bridge opens to form a terminal SH^–^ on Fe2, similar to the cluster model. However, when placing CO at Fe2, the CO surprisingly assumes the bridging position (μ-CO) between Fe6 and Fe2 during the geometry optimization. At the same time, the protonated belt sulfide bridge becomes a terminal SH^–^ on Fe6. The binding energies are −8.3 and −5.1 kcal/mol for the terminal and the bridging CO structure, respectively. Therefore, the binding in the E_1_ QM/MM model is about 7 kcal/mol weaker than in the E_1_ cluster model. The reason behind the less favorable binding appears to be the His195 residue. In the discussion of the E_1_ cluster model, binding CO to either Fe6 or Fe2 has led to an open Fe6/Fe2 bridge, that is a terminal CO and a terminal SH^–^ on neighboring Fe centers. However, a terminal ligand on Fe2 interferes with the His residue in the QM/MM model (cf. E_0_ QM/MM model in Figure 4). Even though the E_1_ QM/MM model with a bridging CO does not have a terminal ligand bound to Fe2, the CO ligand assumes the less favorable bridging binding mode and thus avoids interference with the His residue. This is most likely the reason for the less strong binding in the E_1_ state in the QM/MM model compared to the cluster model. Furthermore, the His residue forms a 1.75 Å hydrogen bond with the CO ligand, somewhat stabilizing the bridging structure. The CO bridge is asymmetric with the Fe–CO bond lengths being 2.022 and 1.795 Å for Fe6 and Fe2, respectively.

The calculated binding energies unfortunately do not paint an unambiguous picture of CO binding to the E_1_ state of FeMoco. While the cluster model indicates a clearly increased binding affinity upon FeMoco reduction and protonation, the QM/MM model shows little change in the binding affinity. The protein environment in the QM/MM model appears to disfavor the structural rearrangements around Fe2 and Fe6, namely, the sulfide bridge opening, leading to weaker binding in the E_1_ state. It is hard to assess whether this rearrangement is similarly prohibited in the real system or whether our methodology fails to capture the conformational flexibility of the protein environment. It seems plausible that upon binding of CO to an E_1_ redox state (as clearly favored in the FeMoco cluster model) the protein environment may conformationally adapt to accommodate the ligand. Any sophisticated conformational change would not be accounted for in our simplistic potential energy surface calculations that are biased toward unbound FeMoco and may require more elaborate free-energy simulations, which are outside the scope of this study.

The calculated CO vibrational frequencies for the E_1_ QM/MM model are 1922 and 1716 cm^–1^ for the terminal and the bridging binding mode, respectively (see Table 2). The frequency for the terminal CO bound to Fe6 is about 45 cm^–1^ lower compared to the E_0_ QM/MM model. This reduction in frequency correlates with an increase in the C–O bond length of 0.004 Å. However, the Fe–CO bond length is 0.02 Å longer in the E_1_ QM/MM model, despite the more activated CO. Notably, the frequency for the bridging CO is 100–250 cm^–1^ lower than all CO-bound QM/MM models discussed so far, allowing for a clear distinction between a terminal and a bridging CO.

**Figure 5:**
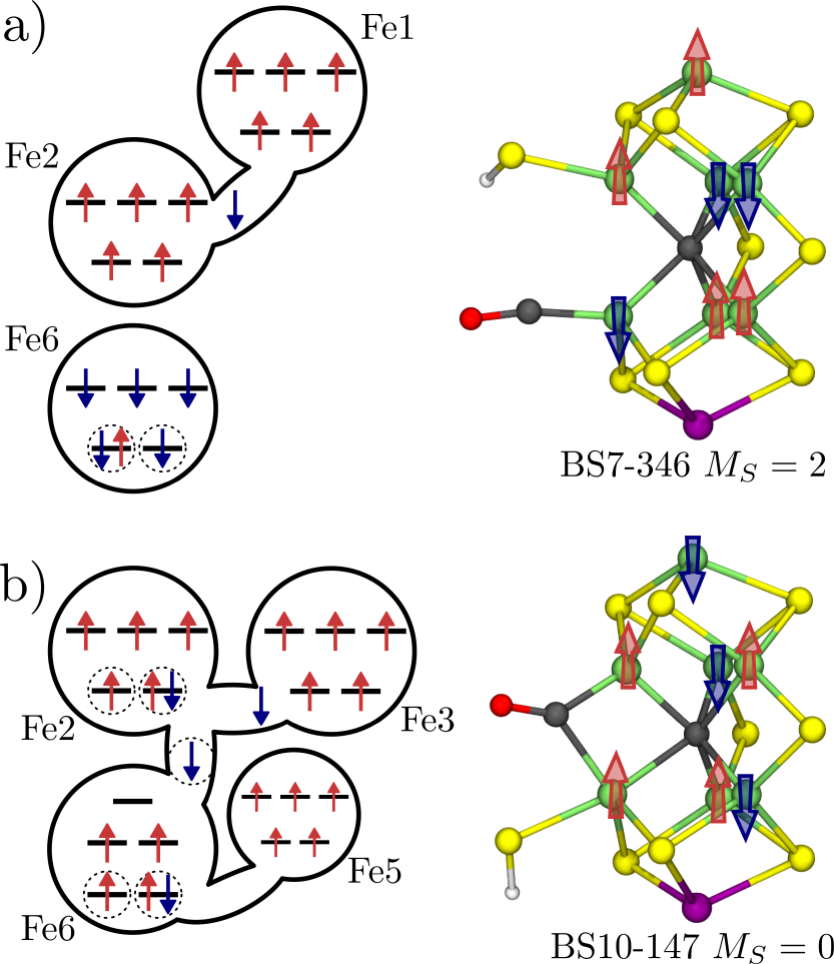
Two CO binding modes in the E_1_ QM/MM model featuring (a) a terminal CO and a terminal SH^–^ and (b) a bridging CO (μ-CO) and a terminal SH^–^. The orbital diagrams show the electron distribution at Fe6 and Fe2 and the involvement of neighboring Fe centers. The dashed circles highlight orbitals that significantly overlap with CO. The large arrows in the FeMoco structures show the local majority spin for each Fe center and therefore visualize the BS determinant.

The electron distribution in the two CO binding modes observed in the E_1_ QM/MM model is shown in Figure 5. For the terminally bound CO (Figure 5a), the additional electron added in the E_0_ to E_1_ redox event localizes on Fe6 and contributes directly to the π back bonding. In contrast to the E_0_ models, CO binding does not induce spin-pairing neither through a local spin flip nor through the localization of a delocalized electron. The CO-bound Fe center in the E_1_ QM/MM model therefore remains a local high-spin center. Nevertheless, according to the QM/MM model, the activation of CO is stronger in the E_1_ state compared to the E_0_ state, even though only three and not four electrons are involved in the π back bonding.

For the bridging CO (Figure 5b), the lowest-energy determinant is BS10-147, and Fe6 and Fe2 are ferromagnetically aligned, which is also the favorable alignment according to the coupling constant in the diamagnetically substituted cofactor μ-CO–[Fe_2_Ga_5_InHS_9_C]^2+^ (*J* = +34 cm^–1^). A total of seven Fe-based orbitals have a strong overlap with the CO π* orbitals (indicated by dashed circles). Using the orbital labels introduced previously for the diamagnetically substituted cofactor (Figure 2c), the delocalized orbital is the bonding linear combination of the out-of-plane *xy* orbitals on Fe6 and Fe2. The doubly occupied orbitals on Fe6 and Fe2, respectively, are the in-plane *yz* orbitals, while the two singly occupied orbitals are the out-of-plane *xy* orbitals. However, a more detailed comparison of the electronic structures of the μ-CO-bound E_1_ QM/MM model, and μ-CO–[Fe_2_Ga_5_InHS_9_C]^2+^ reveals some differences: (i) Fe2 remains a local high-spin center in the full cofactor model but has a local intermediate spin in μ-CO–[Fe_2_Ga_5_InHS_9_C]^2+^. (ii) The full cofactor model features a delocalized electron between Fe6 and Fe2, the electrons in μ-CO–[Fe_2_Ga_5_InHS_9_C]^2+^ are localized on either Fe6 or Fe2. (iii) The orbitals that are preferentially doubly occupied are the in-plane *yz* orbital for the full cofactor model but out-of-plane *xy* for μ-CO–[Fe_2_Ga_5_InHS_9_C]^2+^. These differences can be attributed to the spin–spin interaction between Fe6/Fe2 and the remaining metal centers in FeMoco. For example, (i) illustrates the competition between a local intermediate spin, which strengthens the Fe–CO bond through spin-pairing and a local high-spin, which contributes to the stability of the BS determinant, because a high-spin Fe center exhibits stronger antiferromagnetic coupling with its neighbors.

An interesting observation is related to the local spin state and the coordination number of the Fe centers. All CO-bound Fe centers in the E_0_ models exhibit a local intermediate spin. For the E_1_ models, the only intermediate spin state is observed in the bridging CO structure, in which Fe6 is ligated by both μ-CO and a terminal SH^–^ (Figure 5b). Local intermediate spin states apparently arise when five ligands are bound to an Fe center resulting in an approximately trigonal-bipyramidal coordination. In contrast, local high-spin states are observed for an approximately tetrahedral environment, even if CO is part of the coordinating ligands. Therefore, the appearance of a local intermediate spin appears to be related more to the local geometry and the coordination number than to the nature of the ligand. Considering the competition between on-site spin-pairing and inter-site spin-coupling discussed during the comparison of μ-CO–[Fe_2_Ga_5_InHS_9_C]^2+^ with the full cofactor model, any pentacoordinated Fe center might be unfavorable for FeMoco because its potential local intermediate spin may affect the antiferromagnetic coupling to the remaining metal centers. This important mechanistic detail might be translated to the binding of other substrates, such as acetylene or N_2_.

The calculated vibrational frequencies for terminal and bridging CO in the E_1_ QM/MM model (1922 and 1716 cm^–1^, respectively) are in good agreement with the bands corresponding to loCO_IR,1_ and loCO_IR,2_ in the SF-FT-IR experiment (1904 and 1715 cm^–1^, respectively), indicating the relevance of these coordination geometries to the mechanism of CO inhibition.^38^ The first, transient band (loCO_IR,1_) has been shown to shift by 11 cm^–1^ to lower frequencies with the mutation of the Val70 residue to Ile.^40^ The same mutation in our E_1_ QM/MM model resulted in a decrease of the calculated 1922 cm^–1^ frequency by 17 cm^–1^, further supporting the assignment of a terminal CO at Fe6 to the loCO_IR,1_ species. Furthermore, the calculated frequency of the bridging CO in the E_1_ QM/MM model agrees well with the band observed in loCO_IR,2_. With the apparent necessity of the His195 residue stabilizing the bridging CO, the Fe6/Fe2 pair is the most probable binding site for the bridging CO, consistent with the CO-bound X-ray structure loCO_XRD_. Admittedly, the CO–[FeGa_6_InS_9_C]^0^ model suggests that frequencies below 1800 cm^–1^ could be possible for a terminal CO bound to an Fe^1+^ center in the chemical environment of FeMoco (cf. Table 1). However, the local oxidation state Fe^1+^ was not observed in any of the full cofactor models and appears rather unlikely, considering the flexible charge redistribution within the cofactor, which is possibly facilitated by the highly covalent μ_6_–C^4–^ center.^64^

In the SF-FT-IR experiment, the 1904 cm^–1^ band (loCO_IR,1_) completely converts to the 1715 cm^–1^ band (loCO_IR,2_) within minutes, suggesting that the latter corresponds to a thermodynamic sink. The energies of the terminal and the bridging CO structure in the E_1_ QM/MM model lie merely 3 kcal/mol apart (terminal lower in energy), which does not indicate a preference for either binding mode. The CO-bound X-ray structure (loCO_XRD_) also features a bridging CO between Fe6 and Fe2, but the S2B belt sulfide is missing completely, suggesting sulfide dissociation as a logical pathway for the complete conversion to a bridging CO. The dissociation of SH^–^ from the CO-bound E_1_ QM/MM model is energetically unfavorable, but there are at least two effects that need to be considered in this context. First, the change in entropy is an important contribution to the dissociation free energy, which is not captured by our potential energy surface calculations. Second, the lability of the belt sulfide has been demonstrated for protein-bound FeMoco in which the S2B position was selectively substituted with Se.^97^ Here, in the CO-inhibited form (Se analogue of loCO_XRD_), Se has been shown to further replace the other two belt sulfides S3A and S5A. The high mobility of Se during CO inhibition might indicate a more complex pathway for the sulfide expulsion from the cofactor *via* the S3A and S5A belt positions rather than the simple dissociation of SH^–^ or H_2_S from the S2B belt position. To test such a mechanism would require the simultaneous and accurate treatment of the electronic structure of FeMoco and the conformational flexibility of the protein environment in a combined DFT/molecular dynamics (MD) simulation, which is beyond the scope of this study. Alternatively, sulfide dissociation might not happen in the E_1_ redox state after all, but additional protonation and reduction might be necessary to dissociate the belt sulfide. To summarize, the calculated frequencies in the CO-bound E_1_ QM/MM model are consistent with the SF-FT-IR bands under low CO pressures (loCO_IR,1_ and loCO_IR,2_), but it neither captures the conversion of a supposedly terminal to a bridging CO suggested by the time-dependence of the SF-FT-IR bands nor the dissociation of the belt sulfide indicated by the CO-bound X-ray structure (loCO_XRD_).

Henthorn *et al.* probed the electronic structure of CO-bound FeMoco in the MoFe protein through Se substitution and Se K-edge high-energy resolution fluorescence detected (HERFD) XAS.^64^ Their results are consistent with the Fe3/Fe4/Fe5/Fe7 centers being more oxidized after CO binding compared to the resting state MoFe protein and, therefore, the electron density appears to shift toward the CO binding site. This experimentally observed redox rearrangement for Se-substituted FeMoco is not apparent in the localized orbitals of our CO-bound E_1_ QM/MM model, where the oxidation state of the Fe3/Fe4/Fe5/Fe7 centers is equivalent to the E_0_ QM/MM model. In this context, it should be noted that the spatially resolved anomalous dispersion (SpReAD) refinement of the Fe K-edge XAS of the resting state MoFe protein supports a rather localized electron distribution in FeMoco, with Fe3/Fe7 being more oxidized compared to Fe2/Fe4/Fe5/Fe6.^19^ Meanwhile, the Fe2/Fe3 and Fe6/Fe7 pairs have a formal Fe^2.5+^ oxidation state according to the localized orbital analysis in the E_0_ QM/MM BS7-235 model.^18^ The link between DFT models and experiment is further complicated by the presence of two other low-energy determinants (BS7-346 and BS7-247) in the calculations that change the location of those mixed-valence pairs, meaning that experiments may be measuring a complex average of multiple electronic configurations, making comparison with a single BS determinant insufficient. Additionally vibronic coupling at experimental temperatures may serve to localize electrons in FeMoco. Therefore, the BS-DFT approach is likely insufficient to treat all aspects of the complex electronic structure of FeMoco. Recent advances have enabled the application of multiconfigurational quantum chemical methods to systems that have the size and complexity of FeMoco.^98−100^ It remains to be seen how far these methods can be pushed in the future toward quantitative accuracy.

### Proposed CO Binding Mechanism

**Figure 6:**
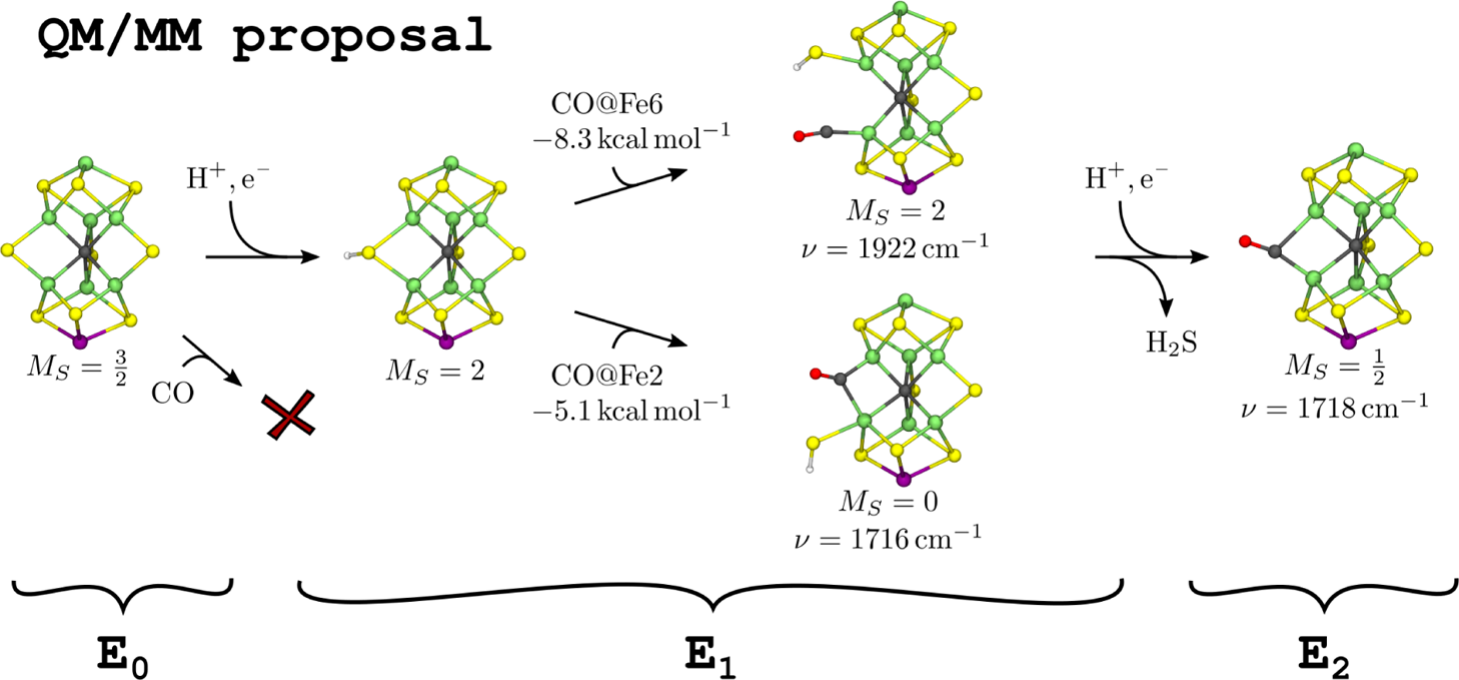
Mechanism for the binding of a single CO molecule to FeMoco based on QM/MM calculations. In the E_0_ state, CO binding is found to be unlikely in the resting state model, consistent with the experiment. In the E_1_ state, the protonated Fe2/Fe6 sulfide bridge spontaneously opens upon CO binding. Two stable structures, one featuring a terminal and one a bridging CO, lie close in energy, and their calculated vibrational frequencies agree well with the experimentally observed SF-FT-IR bands at 1904 and 1715 cm^–1^ (loCO_IR,1_ and loCO_IR,2_, respectively). Alternatively, an additional protonation/reduction event to the E_2_ redox state might be necessary to dissociate SH^–^ from the cofactor, as observed in the X-ray structure (loCO_XRD_). In the QM/MM model, this leads to a semi-bridging CO with a calculated frequency also consistent with the SF-FT-IR species loCO_IR,2_ and an *M*_S_ value consistent with the *S* = 1/2 EPR signal loCO_EPR_.

Finally, we would like to propose the mechanism in Figure 6 for the binding of CO to FeMoco based on our QM/MM model. CO binding to the wild-type MoFe protein generally requires turnover conditions suggesting one or multiple binding E_n_ states with n > 0. Agreeing with this observation, the calculated terminal CO frequencies in the E_0_ QM/MM model are 50–60 cm^–1^ higher than the initial, transient SF-FT-IR band at 1904 cm^–1^ (loCO_IR,1_) suggesting that a more reduced CO-bound FeMoco gives rise to this IR species. The E_1_ QM/MM model features both a terminal and a bridging CO bound to FeMoco, and the calculated CO frequencies, 1922 and 1716 cm^–1^, are consistent with the initial and final SF-FT-IR bands observed under turnover conditions and low CO pressures at 1904 and 1715 cm^–1^, respectively (loCO_IR,1_ and loCO_IR,2_). In both cases, CO binding led to the spontaneous opening of the protonated S2B belt sulfide bridge. However, the CO-bound X-ray structure (loCO_XRD_) indicates that S2B eventually dissociates from FeMoco, and the conversion of the SF-FT-IR species loCO_IR,1_ to loCO_IR,2_ suggests that the latter bridging CO species is a thermodynamic sink. Our QM/MM model does not support sulfide dissociation in the form of SH^–^ to occur at the E_1_ redox level. Starting with an elongated Fe6–SH^–^ bond length of 3.3 Å, the geometry optimization reconverged to the same SH^–^-bound structure. Furthermore, protonation of SH^–^ from homocitrate also did not lead to spontaneous sulfide dissociation. With an initially elongated FeMoco–H_2_S bond the geometry optimization converged to a structure with H_2_S loosely bound in between homocitrate and Gln191, but more than 30 kcal/mol higher in energy. Exploring the dissociation pathways further would most likely require the inclusion of thermodynamic effects in a DFT/MD simulation, which is beyond the scope of this study. Nevertheless, the E_1_ redox state is an even-electron state and can therefore not be responsible for the *S* = 1/2 EPR signal (loCO_EPR_), which is detected in the sample of the CO-bound X-ray structure (loCO_XRD_). This makes the odd-electron E_2_ state a plausible candidate for the EPR signal loCO_EPR_. Therefore, we will briefly explore the properties of a CO-bound E_2_ state in a QM/MM model in which S2B is missing.

We assume for our E_2_ QM/MM model that the additional proton is used to dissociate the S2B belt sulfide from FeMoco in the form of H_2_S, because a quantum refinement study of the X-ray structure loCO_XRD_ suggests that CO-bound FeMoco is not protonated,^52^ and therefore have removed H_2_S completely from the model. CO in the E_2_ QM/MM model forms a semi-bridge between Fe6 and Fe2 (see Figure 6) with Fe–CO bond lengths being 1.765 and 2.250 Å, respectively. The hydrogen bond between CO and His195 is 1.88 Å and therefore only slightly longer (0.13 Å) than in the μ-CO E_1_ QM/MM model. The lowest-energy BS determinant for this binding mode is BS7-235, rather than BS10-147, suggesting that the ferromagnetic alignment of Fe6 and Fe2 is less important with a semi-bridging CO compared to the bridging CO in the E_1_ QM/MM model. The *M*_S_ = 1/2 value of the determinant is consistent with the *S* = 1/2 EPR signal under low CO pressures (loCO_EPR_). Interestingly, the calculated frequency of the semi-bridging CO is with 1718 cm^–1^ virtually identical to the bridging CO in the E_1_ model (1716 cm^–1^). As a consequence, both the E_1_ and the E_2_ QM/MM model are consistent with the frequency observed for the supposedly bridging CO in the SF-FT-IR experiment under low CO pressures (loCO_IR,2_). We wish to emphasize again that this brief exploration merely illustrates that the calculated CO frequencies are similar for an E_1_ model with SH^–^ bound and an E_2_ model with SH^–^ removed. A thorough treatment of any FeMoco redox state requires an in-depth analysis of the electronic structure.

Even though the three classes of nitrogenase are believed to reduce N_2_*via* one unified mechanism,^7^ they exhibit significant differences with respect to CO. Most importantly, in the wild-type Mo nitrogenase, CO merely acts as an inhibitor to N_2_ reduction, while V and Fe nitrogenases reduce CO to hydrocarbons.^15^ On the other hand, Val70 mutants of Mo nitrogenase have the ability to reduce CO, which showcases the crucial role of the protein environment in addition to the electronic structure of the cofactor. It has been reported for V nitrogenase that it can bind CO without turnover conditions,^11,12^ which could be a result of the Fe centers in the V nitrogenase resting state being more reduced compared to Mo nitrogenase.^18,96,101^ However, some controversy exists around this observation and even the nature of the V nitrogenase resting state.^54,102^ Nevertheless, CO binding to V nitrogenase has been shown to elicit similar EPR signals as in Mo nitrogenase (loCO_EPR_ and hiCO_EPR_),^11,12^ as well as analogous X-ray structures (loCO_XRD_ and hiCO_XRD_).^54,103^

In connection with the X-ray structures, it has been proposed that an E_2_ state containing a hydride bridging Fe6 and Fe2 is a prerequisite to CO binding.^54^ Elimination of H_2_ would then lead to an E_0_ redox state with CO bridging Fe6 and Fe2 and supposedly the redox state of the X-ray structure loCO_XRD_. Preliminary calculations for an E_0_ QM/MM model without the bridging sulfide suggest that the CO frequency is more than 100 cm^–1^ higher compared to the E_2_ QM/MM model, a consequence of the two-electron oxidation. The calculated CO frequency of the E_2_ QM/MM model is close to the experimental SF-FT-IR band loCO_IR,2_. This once more emphasizes the importance of applying multiple and ideally orthogonal experimental techniques when characterizing a given nitrogenase species.

## Conclusions

Herein, we have systematically investigated which oxidation and protonation state of FeMoco is required for CO binding. Generally, binding CO to FeMoco in the wild-type MoFe protein requires turnover conditions, while binding CO to solution-extracted FeMoco requires electrochemical reduction to an E_1_-equivalent cofactor charge state. We have calculated the CO binding energy in the E_0_ and E_1_ redox states using both a cluster model and a QM/MM model (see Table 2 and Figure 3). The E_0_ QM/MM model weakly binds CO at Fe6, but the binding energy (Δ*E* = −7.8 kcal/mol) might be insufficient to overcome the primarily translational entropic penalty associated with the complex formation, which is typically estimated at around 10 kcal/mol, based on gas phase statistical mechanics.^104^ The E_1_ cluster model clearly binds CO (Δ*E* = −15.0 kcal/mol), but for the E_1_ QM/MM model (Δ*E* = −8.3 kcal/mol) the binding is only slightly stronger as compared to E_0_. However, with the protein environment in the QM/MM model being biased toward a substrate-free FeMoco, the surrounding residues may not have the necessary conformational flexibility during a simple potential energy surface calculation to accommodate the structural rearrangements such as the opening of the S2B belt sulfide bridge in the context of CO binding. A more complex simulation that includes the dynamics of the protein environment may be needed to give a realistic binding affinity estimate. On the other hand, the scaled calculated vibrational frequency for a terminal CO bound in the E_1_ QM/MM model (1922 cm^–1^) is in good agreement with the initially observed experimental SF-FT-IR species loCO_IR,1_ (1904 cm^–1^)^38^ Therefore, CO might bind already to the E_1_ state.

Furthermore, we have compared Fe6 and Fe2 as potential CO binding sites because the X-ray structure loCO_XRD_ shows CO bridging Fe6 and Fe2. In the E_1_ cluster model, binding to Fe6 is about 4 kcal/mol more favorable compared to Fe2, in line with the more reduced Mo cubane in the substrate-free E_1_ redox state.^20^ In the E_1_ QM/MM model, the scaled calculated vibrational frequency of a terminal CO bound to Fe6 (1922 cm^–1^) agrees well with the loCO_IR,1_ species (1904 cm^–1^), which is the initially appearing species in the SF-FT-IR experiment. Also, the CO frequency of the supposedly loCO_IR,1_ species has been shown to decrease by 11 cm^–1^ in Val70→Ile mutants,^40^ and the same mutation results in a similar decrease (17 cm^–1^) in the E_1_ QM/MM model. In particular, the His195 residue disfavors binding to Fe2, as can be seen by the about 10 kcal/mol stronger binding to Fe6 as compared to Fe2 for CO in the E_0_ QM/MM model (cf. Figure 4). Therefore, the initial CO binding site is most likely Fe6.

In order to separate the influence of the protein environment on CO binding from the intrinsic properties of the cofactor, we have compared a FeMoco cluster model with a QM/MM model. In addition to shielding Fe2 from initial substrate binding, the His195 residue forms a hydrogen bond with the bridging CO in the E_1_ QM/MM model (see Figure 3d). In the E_1_ cluster model, a terminal CO is the lowest-energy binding mode; therefore, the His195 residue appears to be explicitly responsible for stabilizing a bridging CO motif. The scaled calculated frequency of the bridging CO in the E_1_ QM/MM model (1716 cm^–1^) is consistent with the final band at 1715 cm^–1^ observed in the SF-FT-IR experiment under low CO pressures. Furthermore, the CO-bound X-ray structure loCO_XRD_ also features a bridging CO with a hydrogen bond to His195.

The CO-bound E_1_ QM/MM model displays two distinct binding modes: a terminal CO at Fe6 and a CO bridging Fe6 and Fe2. The scaled calculated CO vibrational frequencies (1922 and 1716 cm^–1^, respectively) are in good agreement with the experimental SF-FT-IR species loCO_IR,1_ and loCO_IR,2_ (1904 and 1715 cm^–1^, respectively).^38^ However, the two binding modes are close in energy (3 kcal/mol, terminal more favorable), which is not consistent with the complete conversion of loCO_IR,1_ to loCO_IR,2_ observed under turnover conditions. Alternatively, CO bound to an E_2_ QM/MM model (with the S2B belt sulfide removed to be consistent with the X-ray structure loCO_XRD_) has virtually the same scaled calculated frequency (1718 cm^–1^) as CO bound to the E_1_ QM/MM model, which suggests that both oxidation states would not necessarily be distinguishable in an IR experiment. The *S* = 1/2 signal in loCO_EPR_ can only arise from an odd-electron redox state such as E_2_. Therefore, a parallel IR/EPR study of CO binding to FeMoco could clarify whether the appearance of the 1715 cm^–1^ band in loCO_IR,2_ coincides with the *S* = 1/2 signal in loCO_EPR_.

In our E_1_ QM/MM model, CO binding leads to the spontaneous opening of the protonated S2B belt sulfide bridge to give a terminal SH^–^ ligand. Since practically no alternative substrates or inhibitors are known to interact with the wild-type MoFe protein in the E_0_ state, we speculate that this type of ligand-bound geometry in the E_1_ redox state may represent the initial mode of binding for various other substrates/inhibitors as well. A terminal SH^–^ has also been proposed in other computational studies, for example, as an intermediate during acetylene reduction^105^ and as an intermediate of the more complicated N_2_ reduction pathway.^63,106,107^ The complete dissociation of the S2B belt sulfide is known to occur as part of CO inhibition from X-ray structures.^46,47^ It remains unclear whether sulfide loss from the cofactor is a prerequisite for the catalytic activity of Mo nitrogenase, as has been discussed in the literature, or whether it is simply a byproduct of CO inhibition.^108−111^

## Supporting Information Available

Example input files for diamagnetically substituted cofactors in Figure 2 (folder: diamagnetic); xyz files for substrate-free models and CO-bound models in Figure 3 (folder: geometries, for QM/MM models both the QM and the active region are included)(ZIP)
QM region used in the QM/MM model for MoFe protein; electronic structure of the substrate-free cofactor; energies of other BS determinants; ChemShell setup; localized orbital analysis; coupling constants; metal–metal distance; vibrational frequencies; and Hirshfeld population analysis (PDF)
