# Supplementary material for: Carbon Monoxide Binding to the Iron–Molybdenum Cofactor of Nitrogenase: a Detailed Quantum Mechanics/Molecular Mechanics Investigation
Source: Inorg Chem. 2021 Nov 12;60(23):18031–47. doi: 10.1021/acs.inorgchem.1c02649 (PMC8653219; doi:10.1021/acs.inorgchem.1c02649)
Supplement: Supplementary file 2 — ic1c02649_si_002.pdf [file ic1c02649_si_002.pdf]

Supporting Information
Carbon Monoxide Binding to the Iron–Molybdenum Cofactor of Nitrogenase: a Detailed Quantum Mechanics/Molecular Mechanics Investigation

Contents

1 QM region 2

2 Electronic structure of the substrate-free cofactor 3

3 Energies of other BS determinants 4

4 Chemshell Setup 7

5 Localized orbital analysis 7

6 Coupling constants 11

7 Metal-Metal distance 11

8 Vibrational frequencies 12

9 Hirshfeld Population Analysis 13

1 QM region

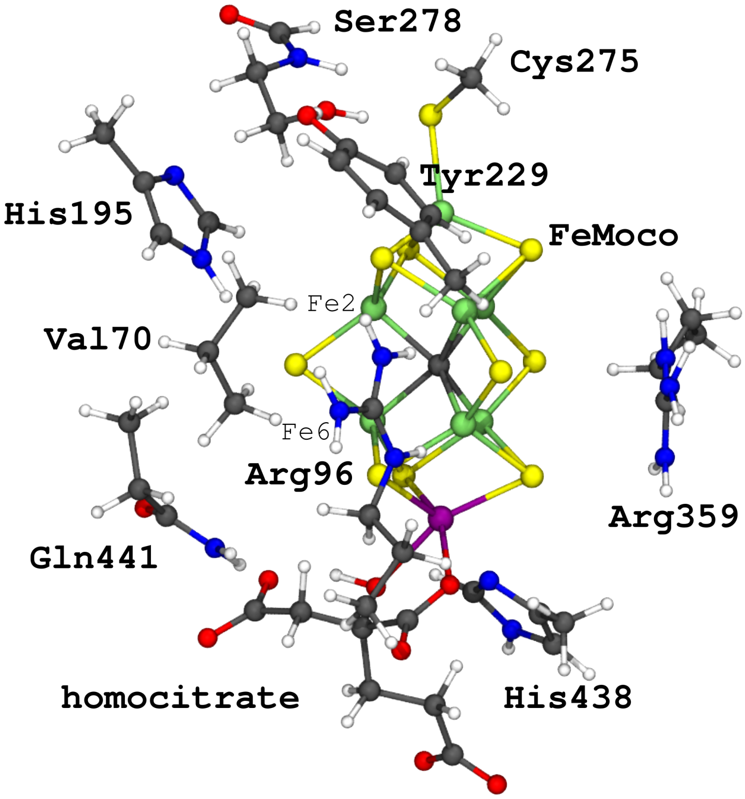

Figure S1 – QM region used in the QM/MM model for MoFe protein. In the E_1 model, the S2B belt sulfide (closest to Val70) is protonated. The residues are labelled according to the crystal structure (PDB ID 3U7Q).

2 Electronic structure of the substrate-free cofactor

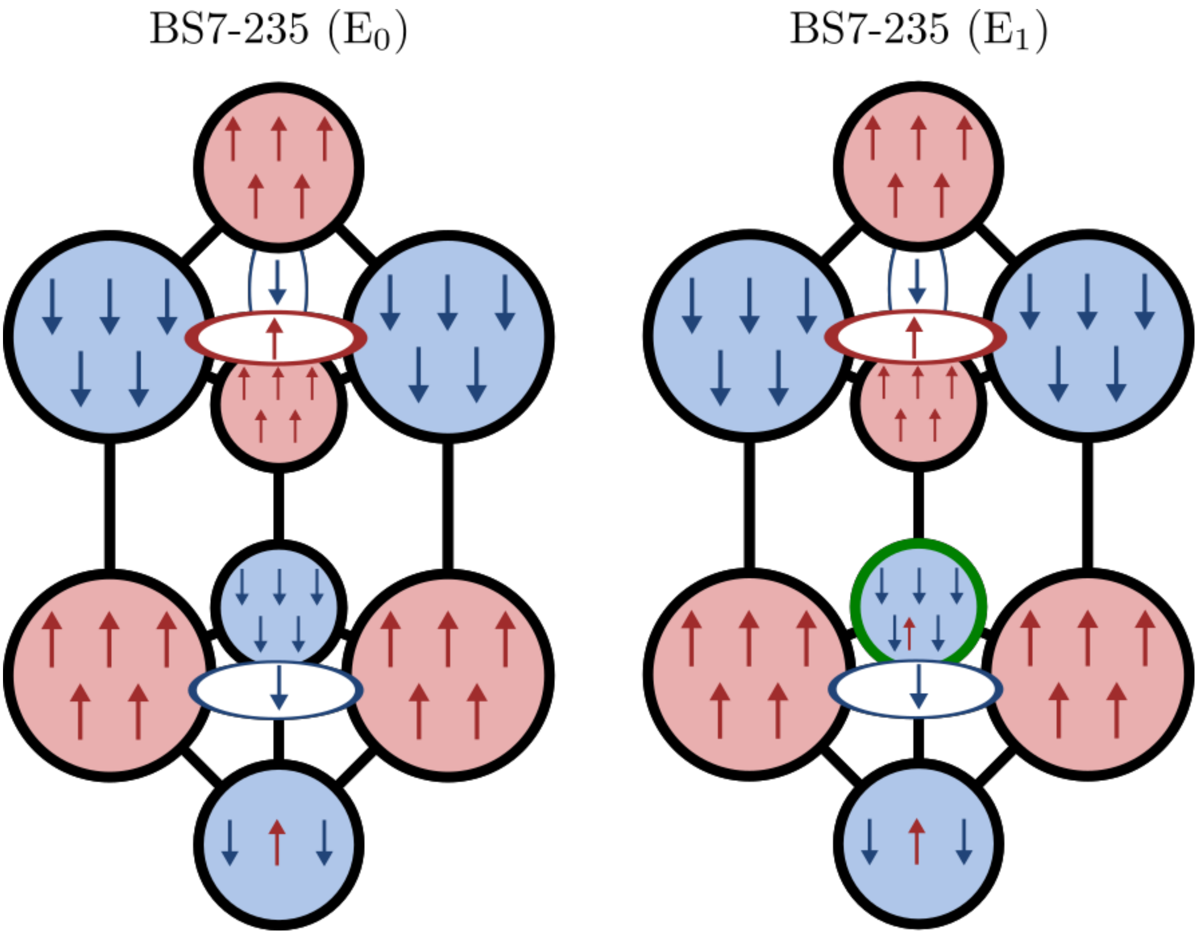

Figure S2 – Electronic structure of the BS7-235 determinant in E_0 ($M_S = \frac{3}{2}$) and E_1 ($M_S = 2$). In E_1, the additional electron is localized on Fe5. The lowest-energy broken-symmetry (BS) determinant in which the electron is localized in the Fe-only cubane lies about 5 kcal/mol higher in energy. The other members of the BS7 class (BS7-247 and BS7-346) are related to BS7-235 by rotation along the pseudo C_3 rotation axis through Fe1 and Mo.

3 Energies of other BS determinants

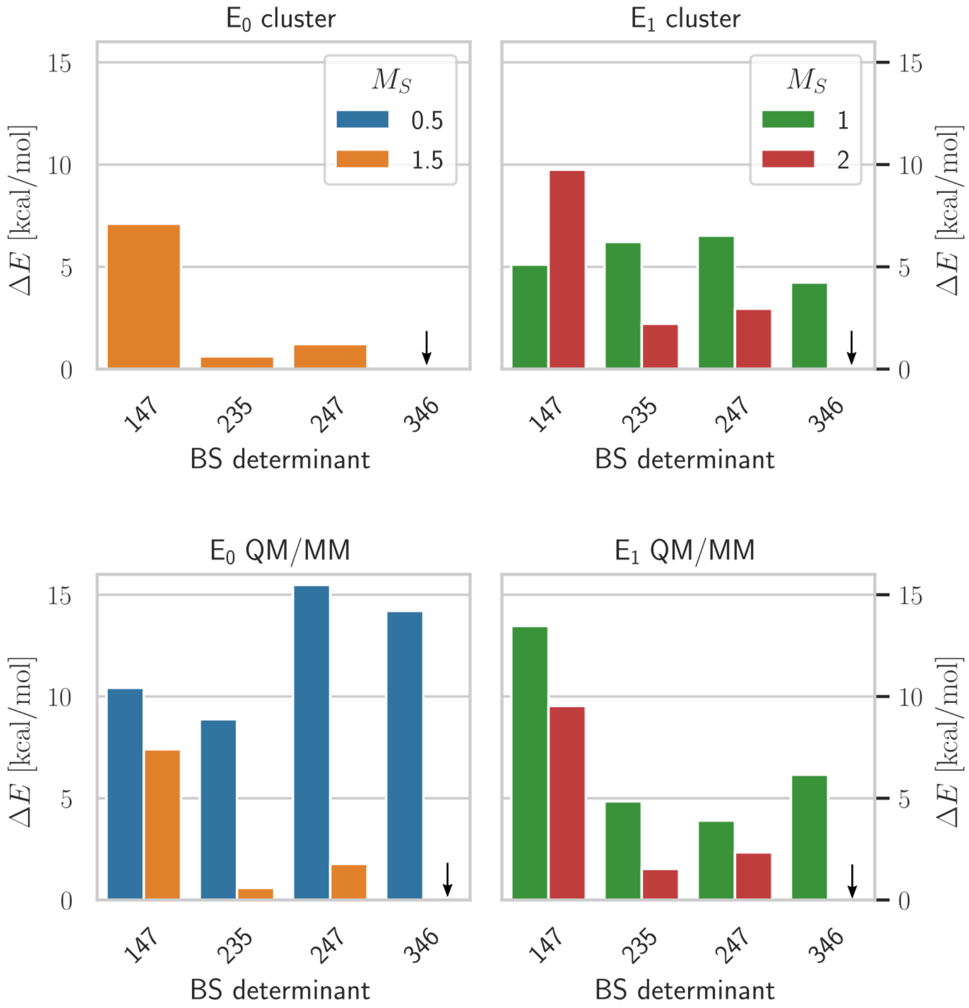

Figure S3 – Relative energies of the optimized, substrate-free models (top: cluster, bottom: QM/MM). The arrow indicates the lowest-energy determinant.

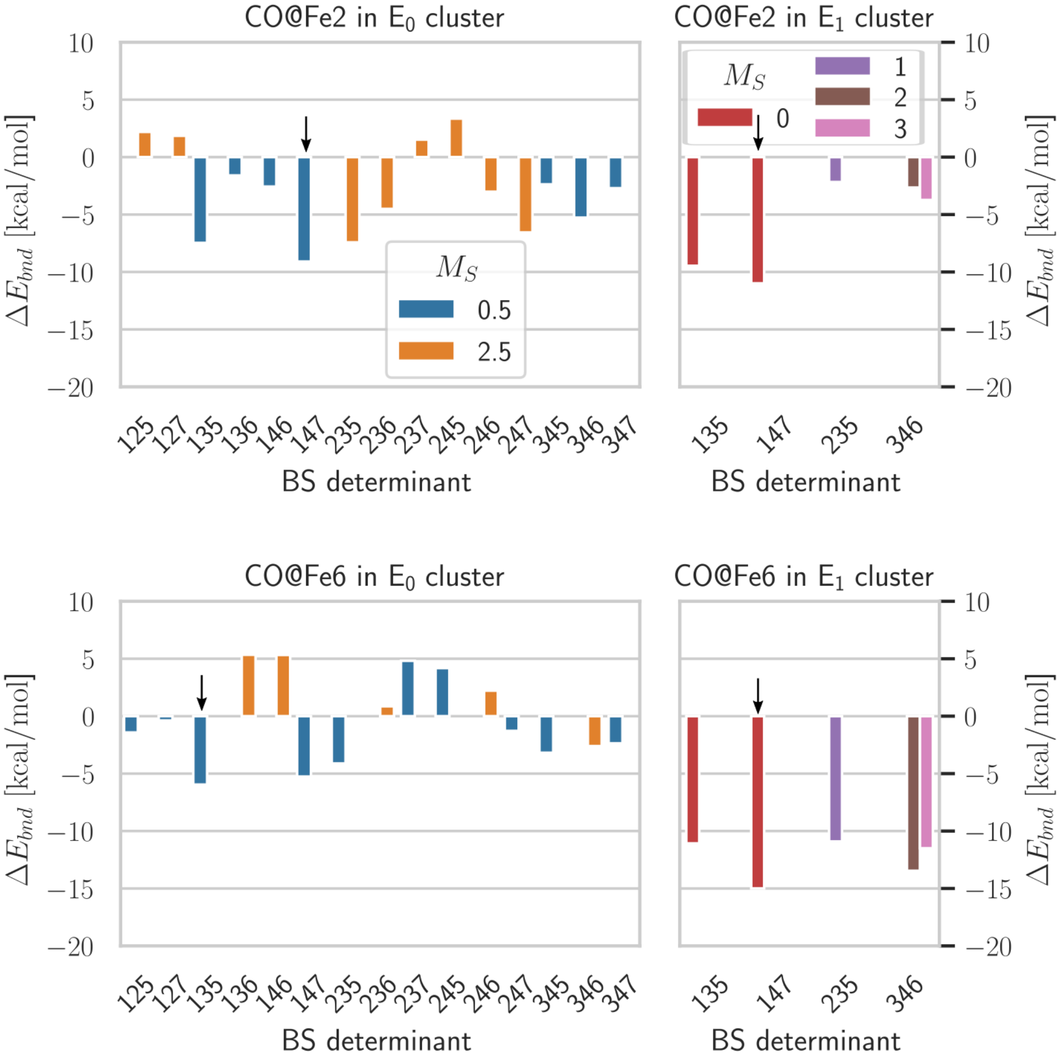

Figure S4 – CO binding energies in the optimized cluster model. A missing bar does not indicate $\Delta E_{bnd} = 0$, but a missing data point. The arrow indicates the lowest-energy determinant. The lowest-energy substrate-free model is shown in Figure S3, respectively.

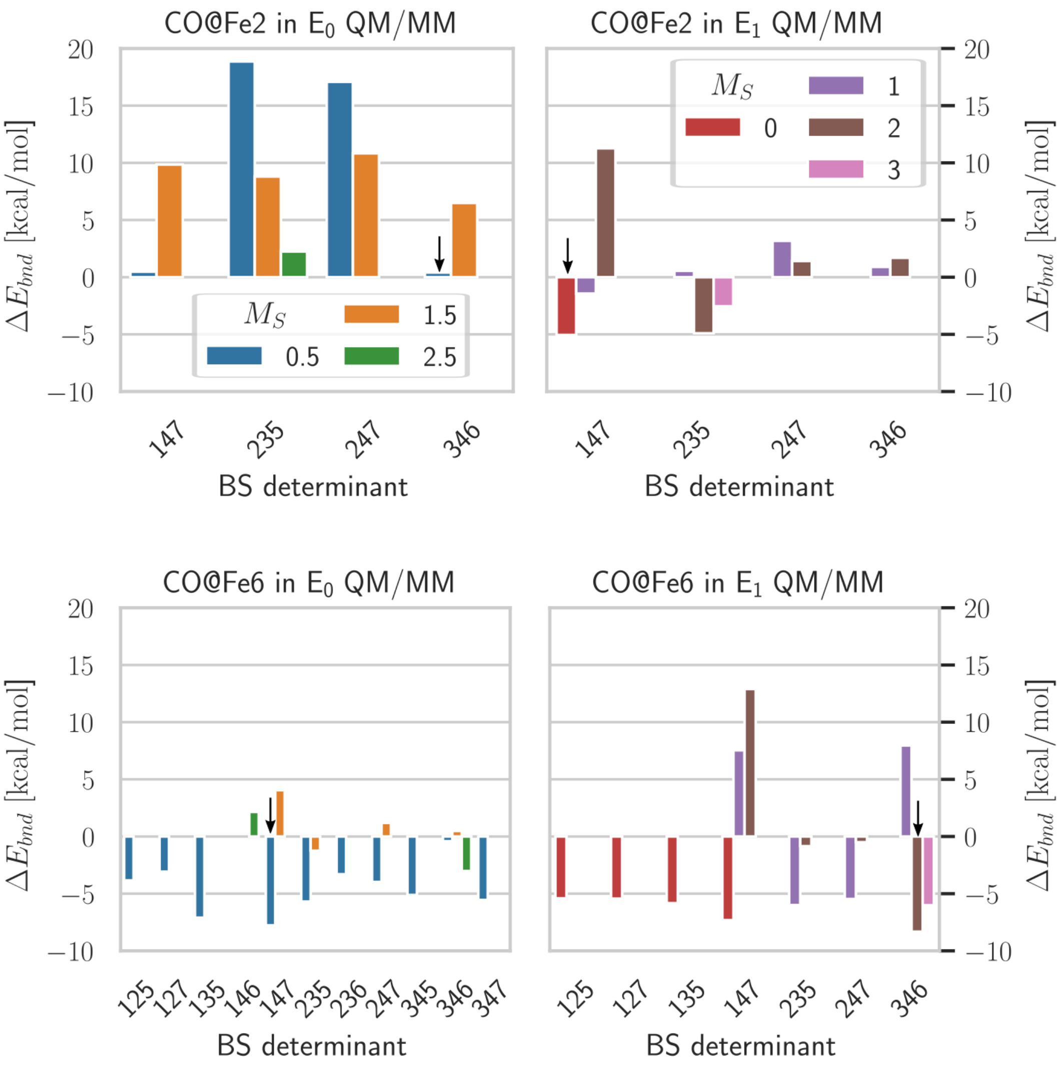

Figure S5 – CO binding energies in the optimized QM/MM model. A missing bar does not indicate $\Delta E_{bnd} = 0$, but a missing data point. The arrow indicates the lowest-energy determinant. The lowest-energy substrate-free model is shown in Figure S3, respectively.

4 Chemshell Setup

The modified Chemshell functions, including the parameter and topology files, can be found under this URL: chemshell-QMMM-protein-setup.

5 Localized orbital analysis

The metal-based localized orbitals are an extremely helpful tool to understand the complex electronic structure of the BS determinants. The chemical environment of the different metal centers vary quite significantly: Fe1 is coordinated by a cysteinate and three μ_3 sulfides, while Fe2 is coordinated by two μ_3 and one μ_2 sulfides. Also, substrate binding changes the chemical environment even more. Certain descriptors of the electronic structure, such as atomic charges, change significantly with the chemical environment. An intuitive picture of the electronic structure in terms of formal oxidation states, such as "this Fe center carries 5 electrons" cannot be derived from those descriptors. Localized orbitals have proven, in our experience, the most reliable method to derive a chemically intuitive representation of the BS determinants for the nitrogenase cofactor. In the localized orbital analysis, the non-metals have the expected formal oxidation state (S^{2-} for the bridging sulfides or C^{4-} for the central carbide), which allows one to focus on the formal metal oxidation states.

A few handy observations can be made when looking at the localized orbitals as tables (e.g. Figure S6): By comparing block structure for the substrate-free E_0 state and the E_1 state, the alignment in the broken-symmetry determinants becomes immediately obvious (blue indicates β spin: Fe3, Fe4, and Fe6 are flipped). Furthermore, one additional row, and therefore electron, is present in the E_1 state, which is located on Fe5. Lighter colors indicate an increasing degree of delocalization (*e.g.* the first β orbital shared between Fe1 and Fe2).

For the substrate-free models, it is sufficient to consider only orbitals largely localized on the metals. However, for CO binding, some of the localized orbitals for the μ−CO models show a strong overlap (see Figure S8). Only those localized orbitals are shown that have a summed weight of 75 % on the atoms selected (metals plus CO, if present).

The workflow to generate the following orbital heat maps from the output of an ORCA calculation *via* the Multwfn program can be adapted to other systems as well and can be found under this URL: multiWFN_analysis

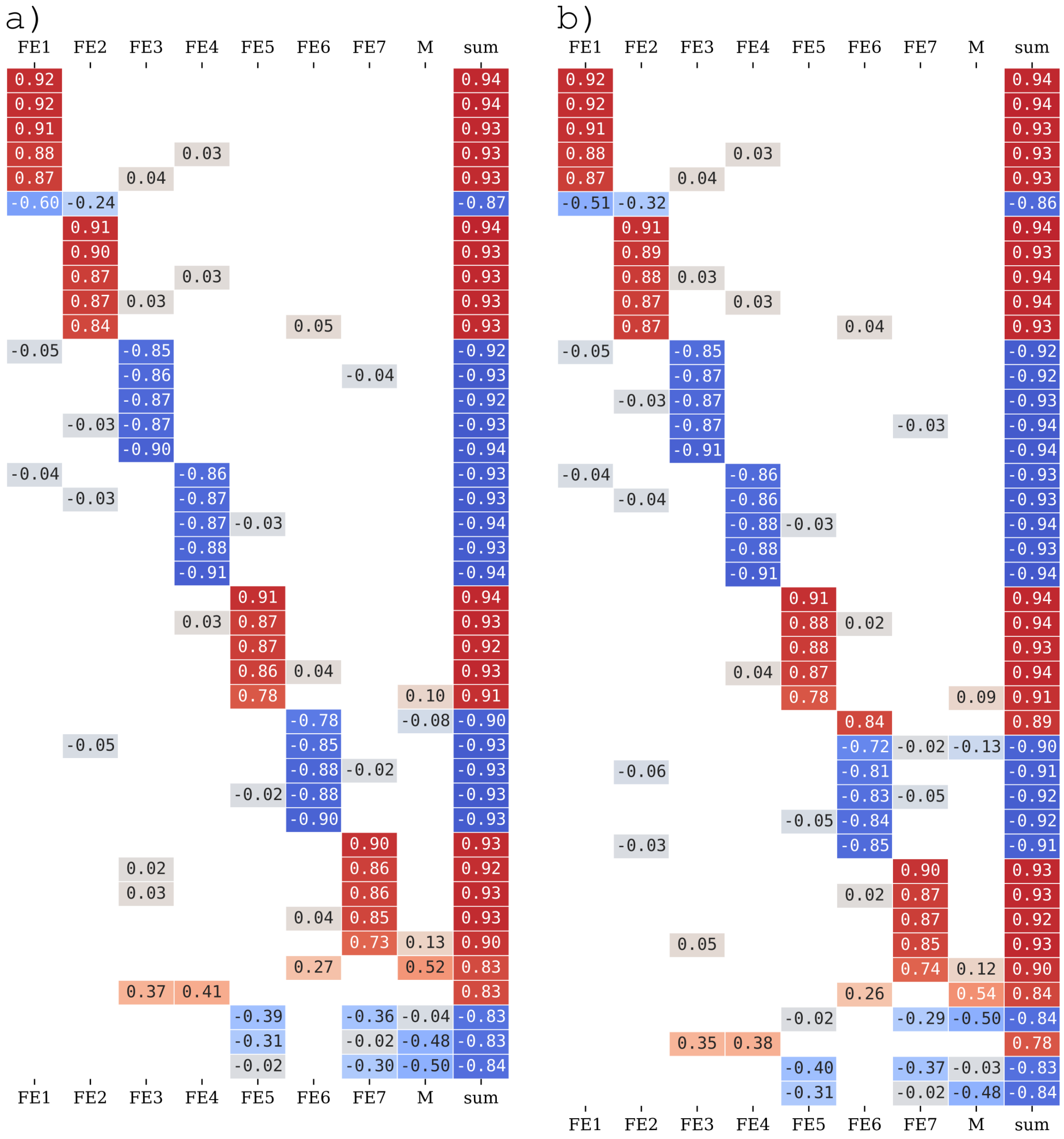

Figure S6 – Metal-based localized orbitals for the substrate-free QM/MM models in the a) E_0 state (BS7-346 $M_S = \frac{3}{2}$) and b) E_1 state (BS7-346 $M_S = 2$). Each row represents an orbitals with a contribution of at $> 75\,\%$ summed over all eight metals (the ordering is arbitrary). Red and positive values indicate α spin orbitals, blue and negative values β spin orbitals. All atomic contributions were determined with the Hirshfeld partitioning scheme. Contributions $< 2\,\%$ are not shown. M represents the heterometal Mo.

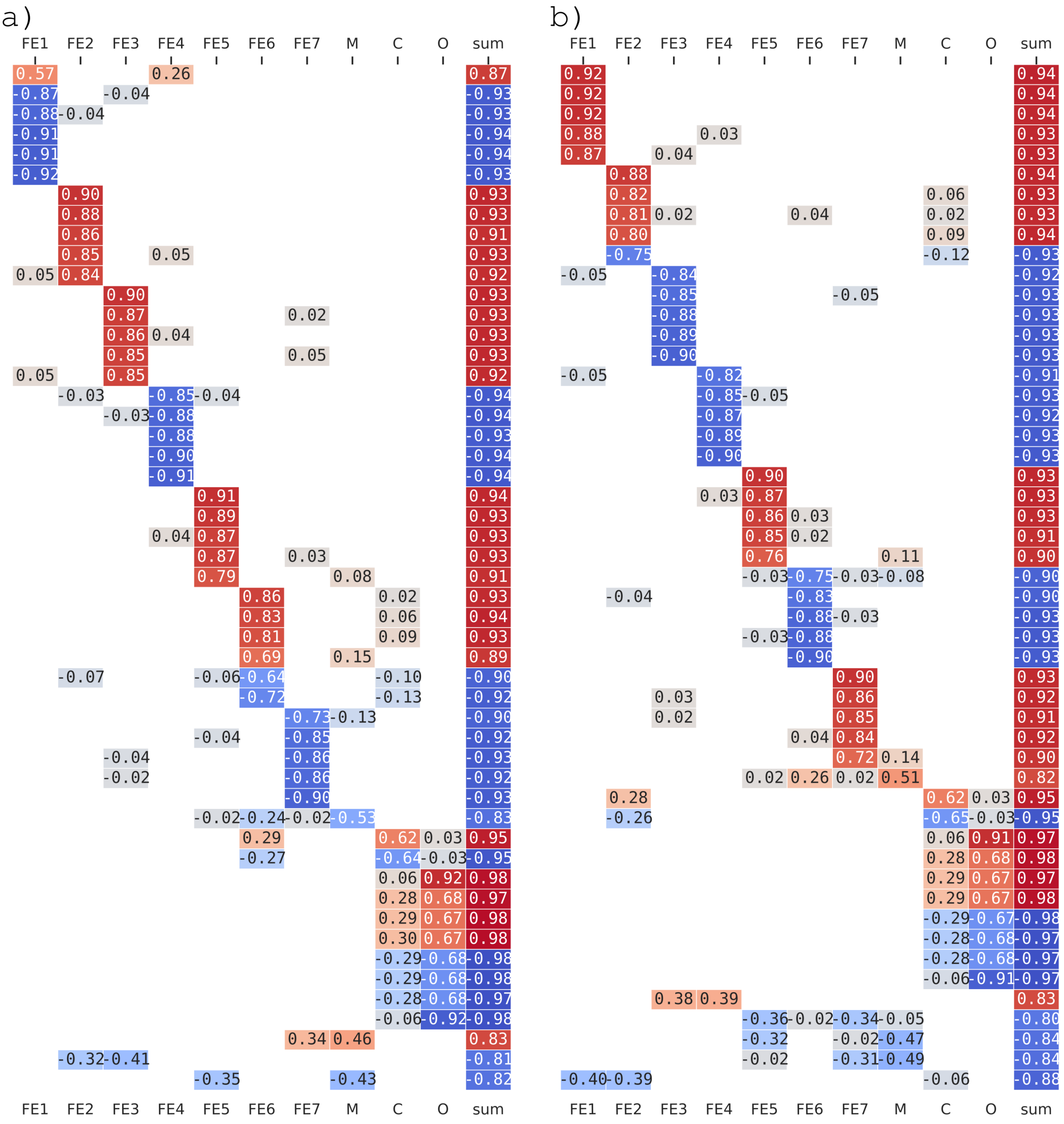

Figure S7 – Metal-based and CO-based localized orbitals for the CO-bound E_0 QM/MM models. a) CO bound to Fe6 (BS10-147 $M_S = \frac{1}{2}$ b) CO bound to Fe2 (BS7-346 $M_S = \frac{1}{2}$). See Figure S6 caption for a more detailed explanation.

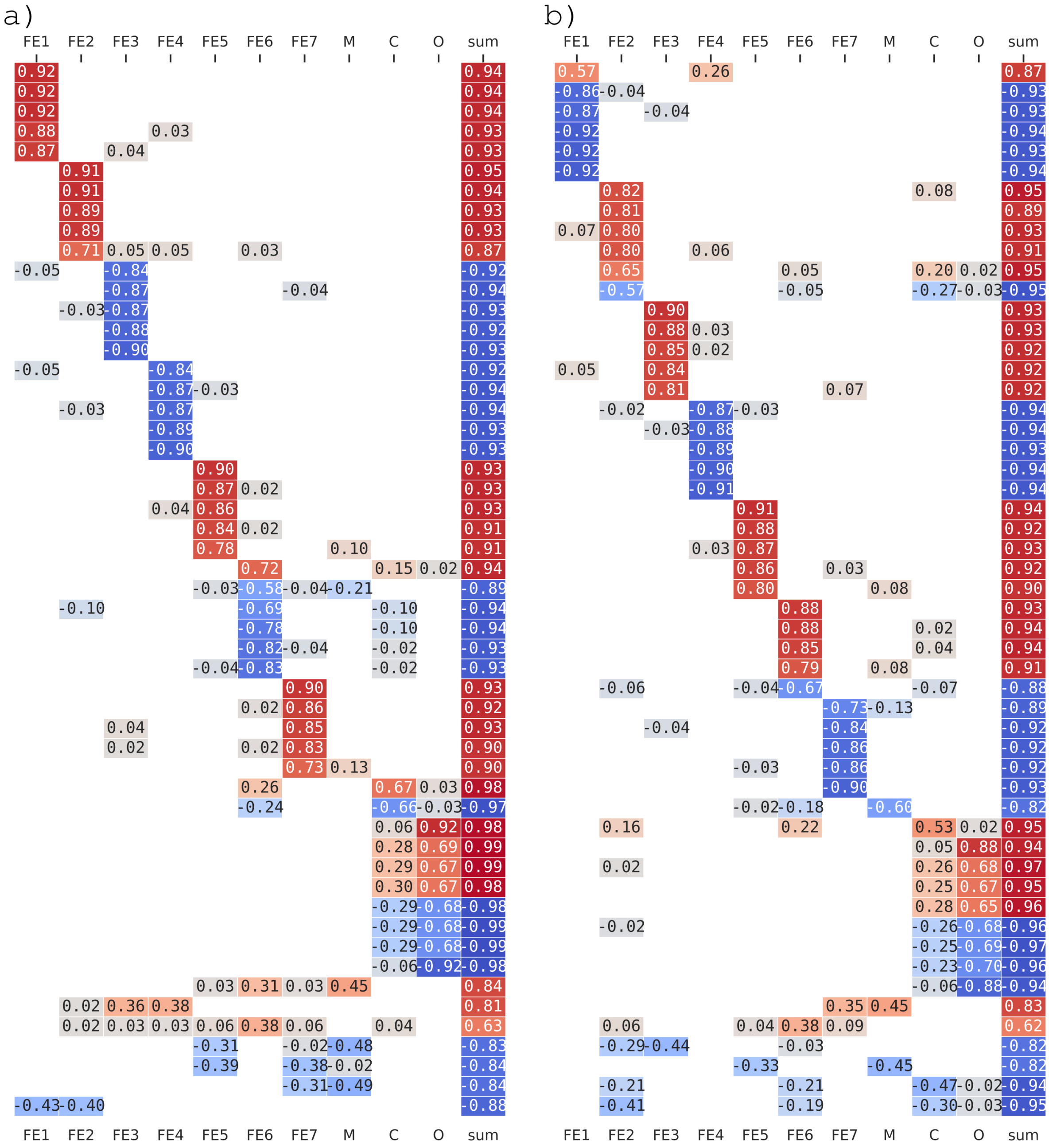

Figure S8 – Metal-based and CO-based localized orbitals for the CO-bound E_1 QM/MM models. a) CO bound to Fe6 (BS7-346 $M_S = 2$ b) CO bridging Fe6 and Fe2 (BS10-147 $M_S = 0$). See Figure S6 caption for a more detailed explanation.

6 Coupling constants

Table S1 – Coupling constants between Fe6 and Fe2 in the diamagnetically substituted cofactors for a mixed-valence $Fe^{2+}Fe^{3+}$ pair. The spin Hamiltonian is given by $H_S = -2JS_AS_B$. The values are obtained through the Yamaguchi projection of the BS determinant. The CO coordinates were optimized only in the cluster model.

CO	model	J cm^{-1}
no CO	cluster	-117.10
CO@Fe6	cluster (ferro opt)	33.62
CO@Fe6	cluster (anti opt)	25.81
μ−CO	E_1 QM/MM (single point)	46.70
μ−CO	E_2 QM/MM (single point)	18.13

7 Metal-Metal distance

Table S2 – Metal-metal distances for substrate-free and CO-bound models. All distances are given in Å.

	E_0 models						E_1 models					
	cluster			QM/MM			cluster			QM/MM		
	free	Fe6	Fe2	free	Fe6	Fe2	free	Fe6	Fe2	free	Fe6	Fe2
FE1/FE2	2.627	2.629	2.728	2.574	2.767	2.616	2.670	2.622	2.624	2.646	2.600	2.568
FE1/FE3	2.661	2.624	2.637	2.669	2.648	2.687	2.621	2.621	2.605	2.612	2.620	2.618
FE1/FE4	2.686	2.660	2.643	2.695	2.648	2.616	2.647	2.636	2.649	2.651	2.636	2.636
FE2/FE6	2.573	2.707	2.668	2.586	2.550	2.566	2.569	2.676	2.630	2.565	2.708	2.483
FE3/FE7	2.609	2.560	2.529	2.614	2.577	2.597	2.574	2.535	2.561	2.572	2.556	2.549
FE4/FE5	2.611	2.557	2.580	2.613	2.596	2.606	2.628	2.584	2.598	2.632	2.610	2.606
FE2/FE3	2.654	2.647	2.868	2.630	2.711	2.553	2.648	2.632	2.784	2.641	2.644	2.527
FE2/FE4	2.654	2.581	2.830	2.632	2.808	2.616	2.629	2.587	2.704	2.611	2.637	2.564
FE3/FE4	2.622	2.626	2.640	2.646	2.640	2.679	2.600	2.599	2.582	2.632	2.611	2.635
FE5/FE6	2.629	2.807	2.560	2.599	2.493	2.664	2.629	2.821	2.625	2.602	2.590	2.811
FE5/FE7	2.559	2.588	2.610	2.561	2.655	2.631	2.574	2.591	2.585	2.580	2.550	2.613
FE6/FE7	2.624	2.796	2.606	2.595	2.557	2.717	2.610	2.771	2.600	2.575	2.577	2.743
Mo/FE5	2.630	2.640	2.649	2.634	2.685	2.649	2.647	2.682	2.618	2.652	2.635	2.699
Mo/FE6	2.709	2.747	2.669	2.662	2.626	2.737	2.721	2.755	2.745	2.663	2.661	2.797
Mo/FE7	2.641	2.664	2.658	2.647	2.655	2.671	2.609	2.632	2.591	2.617	2.586	2.623

8 Vibrational frequencies

Table S3 – As-calculated and scaled CO stretching frequencies. The stretching frequency of gaseous CO is 2143 cm^{-1}. Using the same level of theory as for the cluster/QM/MM models, the calculated frequency of an isolated CO molecule in vacuum is 2181 cm^{-1}. These two values can be used to derive a scaling factor $f = 2143/2181 = 0.9826$. All calculated frequencies reported in the paper were multiplied by f. In the CO$-$[$Fe_2Ga_5InS_9C$] model, the energy of the optimized antiferromagnetically aligned Fe centers is 0.9 kcal/mol higher compared to the ferromagnetically aligned Fe centers.

model		$\nu_{CO,orig}$ [cm^{-1}]	$\nu_{CO,scaled}$ [cm^{-1}]
free CO		2181	2143
		CO$-$[$FeGa_6InS_9C$]	
Fe^{3+}		2013	1978
Fe^{2+}		1918	1884
Fe^{1+}		1803	1772
		CO$-$[$Fe_2Ga_5InS_9C$]	
$Fe^{2+}\uparrow Fe^{3+}\uparrow$		1942	1908
$Fe^{2+}\uparrow Fe^{3+}\downarrow$		1951	1917
		E_0 models	
cluster	Fe6	1944	1910
	Fe2	1929	1896
QM/MM	Fe6	2001	1966
	Fe2	1992	1957
		E_1 models	
cluster	Fe6	1854	1821
	Fe2	1889	1856
QM/MM	Fe6	1956	1922
(Val2Ile)	Fe6	1939	1905
	Fe2/Fe6	1746	1716

Table S4 – Dependence of the calculated CO vibrational frequency on the size of the partial Hessian calculation. Values reported for CO bound to Fe6 in the E_1 QM/MM model (BS7-247 $M_S = 1$).

ν_{CO} [cm^{-1}]	unfrozen atoms
1978.1	CO+Fe
1978.1	CO+Fe+3S+carbide
1977.9	CO+6Fe+Mo+3S+carbide

9 Hirshfeld Population Analysis

Table S5 – Hirshfeld spin populations for substrate-free and CO-bound models.

model		FE1	FE2	FE3	FE4	FE5	FE6	FE7	Mo
					E_0 models				
cluster	free	3.36	3.23	-3.11	-3.11	2.81	-3.00	2.81	-0.24
	Fe6	-3.29	3.19	-3.22	3.09	-2.83	1.56	2.96	-0.43
	Fe2	-3.32	1.95	3.22	-3.25	2.85	2.93	-2.83	-0.40
QM/MM	free	3.30	3.20	-3.04	-3.06	2.84	-2.96	2.72	-0.19
	Fe6	-3.28	3.06	3.02	-3.17	2.99	1.51	-2.75	-0.32
	Fe2	3.34	1.81	-3.08	-3.02	2.72	-2.96	2.64	-0.16
					E_1 models				
cluster	free	3.39	3.22	-3.09	-3.09	2.88	-2.45	2.88	-0.29
	Fe6	-3.35	3.22	3.16	-3.25	2.73	1.07	-2.89	-0.32
	Fe2	-3.35	1.61	2.99	-3.30	2.81	3.00	-2.90	-0.36
QM/MM	free	3.33	3.19	-3.03	-3.05	2.90	-2.38	2.79	-0.25
	Fe6	3.35	3.16	-3.05	-3.08	2.77	-1.88	2.66	-0.36
	Fe2	-3.29	2.11	2.95	-3.22	2.91	2.26	-2.73	-0.47

Table S6 – Hirshfeld charges for substrate-free and CO-bound models.

model		FE1	FE2	FE3	FE4	FE5	FE6	FE7	Mo
					E_0 models				
cluster	free	0.042	0.075	0.054	0.056	0.006	0.056	0.010	0.508
	Fe6	0.030	0.066	0.065	0.050	0.019	-0.016	0.039	0.514
	Fe2	0.046	0.019	0.082	0.064	0.006	0.028	0.026	0.508
QM/MM	free	0.080	0.114	0.090	0.033	0.031	0.087	0.011	0.498
	Fe6	0.079	0.094	0.083	0.040	0.062	0.001	0.024	0.504
	Fe2	0.094	0.030	0.090	0.038	0.017	0.088	0.000	0.494
					E_1 models				
cluster	free	0.045	0.082	0.046	0.048	0.005	-0.009	0.010	0.498
	Fe6	0.049	0.101	0.075	0.072	0.016	-0.060	0.036	0.507
	Fe2	0.044	-0.024	0.057	0.071	0.011	0.058	0.034	0.514
QM/MM	free	0.085	0.106	0.088	0.029	0.034	0.005	0.016	0.491
	Fe6	0.099	0.116	0.084	0.029	0.028	-0.008	0.008	0.492
	Fe2	0.083	0.042	0.087	0.049	0.061	0.037	0.026	0.507
